# Phylogeny and reassessment of the genus *Tolypocladium* s. lato.: Proposing a new genus, three new species and two combinations

**DOI:** 10.3897/mycokeys.141.204222

**Published:** 2026-10-13

**Authors:** Wan-Hao Chen, Chuan-Xuan Xiong, Hui-Lin Shu, Dan Li, Qian Zeng, Jian-Dong Liang, Nalin N Wijayawardene, Yan-Feng Han, Jie-Hong Zhao

**Affiliations:** 1 Center for Mycomedicine Research, Basic Medical School, Guizhou University of Traditional Chinese Medicine, Guiyang 550025, Guizhou Province, China Center for Mycomedicine Research, Basic Medical School, Guizhou University of Traditional Chinese Medicine Guiyang China; 2 Institute of Fungus Resources, Department of Ecology, College of Life Sciences, Guizhou University, Guiyang 550025, Guizhou Province, China Institute of Fungus Resources, Department of Ecology, College of Life Sciences, Guizhou University Guiyang China; 3 Key Laboratory of Microbiome and Infectious Disease Prevention & Control in Guizhou Province, Guiyang 550025, Guizhou Province, China Key Laboratory of Microbiome and Infectious Disease Prevention & Control in Guizhou Province Guiyang China; 4 College of Pharmacy, Guizhou University of Traditional Chinese Medicine, Guiyang 550025, Guizhou Province, China College of Pharmacy, Guizhou University of Traditional Chinese Medicine Guiyang China; 5 Center for Yunnan Plateau Biological Resources, Protection and·Utilization & Yunnan International Joint Laboratory of Fungal Sustainable Utilization in South and Southeast Asia, College of Biology and Food Engineering, Qujing Normal University, Qujing, Yunnan, 655011, China Center for Yunnan Plateau Biological Resources, Protection and·Utilization & Yunnan International Joint Laboratory of Fungal Sustainable Utilization in South and Southeast Asia, College of Biology and Food Engineering, Qujing Normal University Qujing China; 6 High-Value Food from Mushrooms and Bioactive Plants in the Green Economy Value Chain Research Group, The Institute of Biotechnology and Genetic Engineering, Chulalongkorn University, Bangkok, Thailand High-Value Food from Mushrooms and Bioactive Plants in the Green Economy Value Chain Research Group, The Institute of Biotechnology and Genetic Engineering, Chulalongkorn University Bangkok Thailand; 7 Department of Bioprocess Technology, Faculty of Technology, Rajarata University of Sri Lanka, Mihintale 50300, Sri Lanka Department of Bioprocess Technology, Faculty of Technology, Rajarata University of Sri Lanka Mihintale Sri Lanka

**Keywords:** *
Hypocreales
*, morphology, multilocus analysis, phylogeny, taxonomic revision

## Abstract

This study presents a phylogenetic reassessment and taxonomic revision of the genus *Tolypocladium* s. str. in *Ophiocordycipitaceae*. A phylogenetic analysis based on five loci (ITS, LSU, *rpb1*, *rpb2*, and *tef*-1α) was conducted, combined with morphological characters and host associations. Traditionally, *Tolypocladium* species are regarded as polyphyletic, with species distributed across three families of *Hypocreales*: *Ophiocordycipitaceae*, *Cordycipitaceae*, and *Clavicipitaceae*. Consequently, we propose a new genus, *Tolypocladiopsis*, to accommodate *Tolypocladium extinguens* (in *Cordycipitaceae*); describe three new species, *Tolypocladium guizhouense*, *T. tongrenense*, and *T. zongqii*, from Fanjing Mountain, Guizhou Province, China. *Tolypocladium lignicola* is accommodated in *Metapochonia* s. str., and we accordingly introduce *M. lignicola*. Our findings refine the generic delimitation of *Tolypocladium*, resolve long-standing taxonomic ambiguities, underscore the misleading nature of morphological convergence in hypocrealean anamorphs, and advocate for an integrative taxonomy that combines molecular phylogenetics with detailed morphological examination.

## Introduction

The genus *Tolypocladium* W. Gams was established by Gams (1971) to accommodate three soil-inhabiting anamorphic species: *T. cylindrosporum* W. Gams, *T. geodes* W. Gams, and *T. inflatum* W. Gams (the type species). Morphologically, the genus is characterized by sparingly branched conidiophores, swollen phialides with distinctly bent necks, and one-celled hyaline conidia aggregated in slimy heads (Gams 1971, 1980; Bissett 1983). For decades, the taxonomy of *Tolypocladium* relied primarily on morphological characteristics and host associations. However, with the advent of molecular phylogenetics, the genus has undergone substantial revisions. Hodge et al. (1996) first linked the anamorph *T. inflatum* to the sexual morph *Cordyceps subsessilis* Petch.

Subsequently, Sung et al. (2007) introduced the genus *Elaphocordyceps* G.H. Sung & Spatafora to accommodate sexual species parasitizing *Elaphomyces* (false truffles) and some arthropods, and linked it to *Tolypocladium* anamorphs based on multigene phylogeny. Following the “One Fungus-One Name” (1F1N) principle, Quandt et al. (2014) synonymized *Elaphocordyceps* and *Chaunopycnis* W. Gams under *Tolypocladium*, as the latter is the oldest and most widely used name. Thus, *Tolypocladium* is currently accepted within the family *Ophiocordycipitaceae (Hypocreales)*, comprising both sexual and asexual morphs (Sung et al. 2007; Quandt et al. 2014).

Recent studies have significantly advanced our understanding of species diversity within *Tolypocladium*. Yu et al. (2021) provided a comprehensive review of the genus and described a new species, *T. inusitaticapitatum* F.M. Yu, Yu, Q. Zhao & K.D. Hyde, from Southwest China based on morphology and multigene phylogeny. Dong et al. (2022) further revised the genus using a five-locus dataset and described three additional new species: *T. pseudoalbum* Hong Yu bis, Yao Wang & Q.Y. Dong, *T. subparadoxum* Hong Yu bis et al., and *T. yunnanense* Hong Yu bis et al. These studies collectively recognized 40 species in *Tolypocladium*, with a key provided for species identification (Yu et al. 2021; Dong et al. 2022). Eight additional species were subsequently reported (Das et al. 2023; Soares et al. 2023; Tan and Shivas 2023; Crous et al. 2024; Song et al. 2024; Li et al. 2025; Lim et al. 2025). Currently, *Tolypocladium* comprises 48 species, of which only nine have been reported from China (Li et al. 2025).

Despite these advances, the generic delimitation of *Tolypocladium* remains problematic. Molecular evidence has repeatedly suggested that *Tolypocladium* may be polyphyletic as currently circumscribed. For instance, *Tolypocladium extinguens* Samson & Soares, a pathogen of the New Zealand glowworm (*Arachnocampa luminosa* Skuse), has been shown to cluster outside the *Tolypocladium* s. str. in preliminary phylogenetic analyses (Dong et al. 2022). Therefore, the genus *Tolypocladium* requires further revision to establish its generic boundaries.

In the present study, we aim to (1) perform a multilocus phylogenetic analysis of *Tolypocladium* and its relatives using an expanded taxon sampling; (2) propose a new genus and new combinations to accommodate taxa that are phylogenetically distinct from *Tolypocladium* s. str.; (3) describe new species discovered from *Cordyceps* sp. and insect substrates.

## Materials and methods

### Specimen collection, isolation, and maintenance

The specimens (i.e., dead insects infected with fungi) were collected from Fanjing Mountain, Jiangkou County, Tongren City, Guizhou Province. The samples were placed in sterile bags with collection details (Rathnayaka et al. 2025), kept separately in an ice box, and transported to the laboratory. The specimens were kept in the refrigerator at 4 °C until they were processed further. A section of the mycelium, or sclerotium was excised from the specimen and positioned on plates containing potato dextrose agar (PDA) or modified PDA by the addition of 1% w/v peptone containing 0.1 g/l streptomycin and 0.05 g/l tetracycline (Chen et al. 2019; Fu et al. 2024; Tarafder et al. 2024). Once fungal colonies appeared on the plated samples, a section of mycelium from the colony’s edge was moved to fresh agar plates and incubated at 25 °C for 14 days with a 12-hour light and 12-hour dark cycle (Zou et al. 2010; Xie et al. 2024a, 2024b). The holotypes and ex-types were stored at the Institute of Fungus Resources, Guizhou University, previously known as the Herbarium of Guizhou Agricultural College (code: GZAC), Guiyang City, Guizhou, China. Index Fungorum numbers are given as described in Index Fungorum (https://www.indexfungorum.org/names/Registration.asp, access 24^th^ September 2026).

### Morphological studies and cultural characteristics

The features of the colonies were assessed on PDA cultures kept at 25 °C for 14 days, noting the growth rate, presence of octahedral crystals, and the colors of the colonies on both the surface and reverse sides. A few mycelia were taken from the colony and placed in lactophenol cotton blue or 20% lactic acid solution to examine microscopic features, and the asexual morphological traits (e.g., conidiophores, phialides or conidiogenous cells, conidia) were observed and measured using a Leica DM4 B microscope.

### DNA extraction, Polymerase Chain Reaction (PCR) amplification and sequencing

A fungal genomic DNA extraction kit (DP2033, BioTeke Corporation) was employed for DNA extraction, following the protocol described by Liang et al. (2011). The extracted DNA was stored at –20 °C for subsequent use. PCR amplification of the target genetic loci was carried out using the following primer pairs: ITS4/ITS5 (amplifying the internal transcribed spacer region, ITS) (White et al. 1990); LR0R/LR5 (targeting the 28S large subunit ribosomal gene, LSU) (Vilgalys and Hester 1990); CRPB1/RPB1Cr (targeting the RNA polymerase II largest subunit gene, *rpb1*) (Castlebury et al. 2004); fRPB2-5F/fRPB2-7cR (targeting the RNA polymerase II second largest subunit gene, *rpb2*) (Liu et al. 1999); and 983F/2218R (amplifying the translation elongation factor 1 alpha gene, *tef*-1α) (Castlebury et al. 2004). The thermal cycling conditions for PCR amplification were based on the methodology outlined by Chen et al. (2021a). The resulting PCR products were purified and sequenced by Sangon Biotech (Shanghai) Co., Ltd. All the used and newly generated sequences were deposited into GenBank, and the corresponding accession numbers were obtained (Table 1).

**Table 1.** List of species, strain numbers and GenBank accession numbers of sequences used in this study.

**Species**	**Strain number**	**GenBank Accession No**.					**Reference**
		**ITS**	**LSU**	***rpb1***	***rpb2***	***tef-*1α**	
*Aciculosporium oplismeni*	MAFF 246966	NR_172530	LC571760	-	LC572054	LC572040	Tanaka et al. 2021
*Aciculosporium take*	MAFF 241224	LC571753	LC571753	-	KP689511	LC572034	Tanaka et al. 2021
*Akanthomyces aculeatus*	HUA 186145^T^	-	MF416520	-	-	MF416465	Kepler et al. 2017
*Akanthomyces aculeatus*	HUA 772	-	KC519370	-	-	KC519366	Sanjuan et al. 2014
*Albacillium hingganense*	CCTCC M 20232069^T^	-	OR740566	OR769082	OR769081	MN065771	Ding et al. 2024
*Arachnidicola sulphurea*	TBRC 7248^T^	NR_164419	MF140722	MF140787	MF140812	MF140843	Mongkolsamrit et al. 2018
*Arachnidicola sulphurea*	TBRC 7249	MF140757	MF140721	MF140786	MF140734	MF140842	Mongkolsamrit et al. 2018
*Ascopolyporus polychrous*	PC 546	-	DQ118737	DQ127236	-	DQ118745	Chaverri et al. 2005
*Ascopolyporus albus*	BCC 48975^T^	OL331502	OL322048	OL322056	OL322065	OL322035	Thanakitpipattana et al. 2022
*Ascopolyporus albus*	BCC 48976	OL331503	OL322049	OL322057	OL322066	OL322036	Thanakitpipattana et al. 2022
*Atkinsonella hypoxylon*	B4728	-	-	-	KP689514	KP689546	Quandt et al. 2014
*Balansia henningsiana*	GAM16112	-	AY545727	AY489643	DQ522413	AY489610	Castlebury et al. 2004; Spatafora et al. 2007
*Beauveria bassiana*	ARSEF 1564^T^	HQ880761	-	HQ880833	HQ880905	HQ880974	Rehner et al. 2011
*Beauveria bassiana*	ARSEF 1478	AY531981	-	HQ880836	HQ880908	AY531890	Rehner et al. 2011; Rehner and Buckley 2005
*Blackwellomyces cardinalis*	OSC 93610	JN049843	AY184963	EF469088	EF469106	EF469059	Sung et al. 2007; Sung and Spatafora 2004
*Blackwellomyces cardinalis*	OSC93609^T^	NR_159788	AY184962	DQ522370	DQ522422	DQ522325	Sung et al. 2007; Sung and Spatafora 2004
*Blackwellomyces changningensis*	YFCC 9250^T^	PP568073	OR492316	OR588131	OR637359	OR608508	Ma et al. 2024
*Blackwellomyces lateris*	MFLU 18-0663^T^	MK086059	MK086061	MK084615	MK079354	MK069471	Hyde et al. 2019
*Blackwellomyces roseostromatus*	BCC 91358^T^	MT000697	MT003033	MT017805	MT017824	MT017845	Mongkolsamrit et al. 2020
*Bhushaniella rubra*	BCC 47541^T^	OQ892128	OQ892133	OQ914431	OQ914433	OQ914428	Mongkolsamrit et al. 2023
*Bhushaniella rubra*	BCC 47542	OQ892129	OQ892134	OQ914432	OQ914434	OQ914429	Mongkolsamrit et al. 2023
*Calcarisporium cordycipiticola*	CGMCC 3.17905^T^	KT944999	KX442599	-	KX442594	KX442593	Sun et al. 2017
*Calcarisporium cordycipiticola*	CGMCC 3.17904	KT945001	KX442604	-	KX442607	KX442605	Sun et al. 2017
*Claviceps purpurea*	GAM 12885	-	AF543789	-	DQ522417	AF543778	Currie et al. 2003; Spatafora et al. 2007
*Claviceps purpurea*	SA cp11	-	EF469075	EF469087	EF469105	EF469058	Sung et al. 2007
*Collarina aurantiaca*	CBS 110646	OQ429535	OQ055447	-	-	-	Hou et al. 2023
*Collarina aurantiaca*	CBS 138274	-	MH877656	-	OQ560707	-	Hou et al. 2023; Vu et al. 2019
*Commelinaceomyces aneilematis*	MAFF 246963	-	LC474617	LC474626	-	LC474623	Tanaka et al. 2020
*Conoideocrella luteorostrata*	NHJ 11343	-	EF468850	EF468906	-	EF468801	Sung et al. 2007
*Conoideocrella luteorostrata*	NHJ 12516	-	EF468849	EF468905	EF468946	EF468800	Sung et al. 2007
*Corallocytostroma ornithocopreoides*	WAC 8705	-	-	-	LT216620	LT216546	Píchová et al. 2018
*Cordyceps militaris*	OSC 93623	JN049825	AY184966	DQ522377	AY545732	DQ522332	Sung et al. 2007
*Corniculantispora psalliotae*	CBS 532.81^T^	MH861374	AF339560	EF469096	EF469112	EF469067	Sung et al. 2001, 2007
*Corniculantispora psalliotae*	CBS 505.48	MH856448	MH867993	-	-	-	Sung et al. 2001
*Corpulentispora magnispora*	CGMCC 3.19304^T^	MK329102	MK329007	-	MK335985	MK336037	Zhang et al. 2021
*Corpulentispora magnispora*	LC12469	MK329103	MK329008	-	MK335986	MK336038	Zhang et al. 2021
*Dimorphspora sinensis*	ZY06451^T^	PV082729	PV082846	-	PV171185	PV171249	Chen et al. 2025
*Dimorphspora sinensis*	ZY06452	PV082730	PV082847	-	PV171186	PV171250	Chen et al. 2025
*Drechmeria balanoides*	CBS 250.82^T^	MH861495	AF339539	DQ522388	DQ522442	DQ522342	Sung et al. 2001; Spatafora et al. 2015
*Drechmeria campanulata*	IMI 356051^T^	AJ292416	AF339543	-	-	-	Zare et al. 2000; Sung et al. 2001
*Drechmeria panacis*	CBS 142798^T^	-	MF588897	-	-	MF614144	Yu et al. 2018
*Drechmeria sphaerospora*	CBS 522.80^T^	AJ292413	AF339541	-	-	-	Zare et al. 2000; Sung et al. 2001
*Drechmeria zeospora*	CBS 335.80^T^	MH861269	AF339540	EF469091	EF469109	EF469062	Sung et al. 2001, 2007
*Dussiella tuberiformis*	ATCC 201937	-	JQ257009	JQ257015	-	-	Kepler et al. 2012b
*Engyodontium aranearum*	CBS 309.85	JN036556	AF339526	DQ522387	DQ522439	DQ522341	Sung et al. 2001; Spatafora et al. 2007
*Ephelis tripsaci*	CBS 857.72	KP859042	KP858978	-	-	-	Hernández-Restrepo et al. 2016
*Epichloe elymi*	C. SchardI 760	-	AY986924	DQ000352	-	AY986951	Chaverri et al. 2005
*Epichloe typhina*	ATCC 56429	JN049832	-	AY489653	DQ522440	AF543777	Chaverri et al. 2005
*Flavocillium acerosum*	CBS 418.81^T^	NR_111268	KM283786	KM283832	KM283852	KM283810	Wang et al. 2020
*Flavocillium bifurcatum*	YFCC 6101^T^	MN576833	MN576781	MN576841	MN576897	MN576951	Wang et al. 2020
*Gamszarea humicola*	CGMCC 3.19303^T^	NR_172830	NG_075268	-	MK335979	MK336027	Zhang et al. 2021
*Gamszarea wallacei*	CBS 101237^T^	NR_111267	NG_042398	EF469102	EF469119	EF469073	Sung et al. 2007
*Gamszarella antillata*	CBS 350.85^T^	NR_111097	AF339536	DQ522396	DQ522450	DQ522350	Spatafora et al. 2007
*Gamszarella buffelskloofina*	CBS 150062^T^	OR680769	OR717025	-	OR683726	OR683715	Zhang et al. 2021
*Gibellula leiopus*	BCC 16025	-	MF416548	MF416649	-	MF416492	Kepler et al. 2017
*Gibellula longispora*	NHJ 12014	-	-	EU369055	EU369075	EU369017	Johnson et al. 2009
*Gibellula nigeli*	NHJ 10808^T^	-	EU369035	EU369056	EU369076	EU369018	Johnson et al. 2009
*Gibellula nigeli*	BCC47555	MH532885	-	MH521804	-	MH521897	Kuephadungphan et al. 2022
*Hantamomyces aloidendri*	CBS 146814^T^	MW175348	MW175388	-	-	-	Crous et al. 2020
*Harposporium anguillulae*	ARSEF 5407	-	AY636080	-	-	-	Chaverri et al. 2005
*Harposporium harposporiferum*	ARSEF 5472^T^	-	AF339519	DQ127238	-	DQ118747	Chaverri et al. 2005; Sung et al. 2001
*Harposporium helicoides*	ARSEF 5354	-	AF339527	-	-	-	Sung et al. 2001
*Helicocollum surathaniensis*	BCC34463	-	KT222328	-	-	KT222336	Luangsa-ard et al. 2017a
*Helicocollum surathaniensis*	BCC34464^T^	-	KT222329	-	-	KT222337	Luangsa-ard et al. 2017a
*Hemileiophthora nodosa*	COAD 3258^T^	-	PQ962830	PV362872	-	PV362921	Colmán et al. 2025
*Hemileiophthora nodosa*	COAD 3259	-	PQ962831	PV362873	PV987262	PV362922	Colmán et al. 2025
*Hevansia arachnophilus*	NHJ 10469	-	EU369031	EU369047	-	EU369008	Johnson et al. 2009
*Hevansia nelumboides*	BCC 41864	-	JN201873	-		JN201867	Kobmoo et al. 2023
*Hevansia novoguineensis*	CBS 610.80^T^	NR_169678	NG_070837	-	MH521844	MH521885	Mongkolsamrit et al. 2022
*Hevansia novoguineensis*	BCC 47881	JX192685	MH394650	MH521807	MH521845	MH521886	Mongkolsamrit et al. 2022
*Heteroepichloe bambusae*		-	MK691595	-	-	-	Geng et al. 2020
*Hirsutella cryptosclerotium*	ARSEF 4517^T^	KM652157	KM652109	KM652032	-	KM651992	Simmons et al. 2015
*Hirsutella guyana*	ARSEF 878	KM652158	KM652111	KM652035	-	KM651994	Simmons et al. 2015
*Hirsutella illustris*	ARSEF 5539	KM652160	KM652112	KM652037	-	KM651996	Simmons et al. 2015
*Hyperdermium bertonii*	AF242354	-	AF242354	-	-	-	Sullivan et al. 2000
*Hypocrella discoidea*	BCC2097	-	-	DQ000346	-	AY986945	Chaverri et al. 2005
*Hypocrella limushanensis*	YHH 2303015^T^	-	OR828401	OR837107	-	OR832089	Wang et al. 2025
*Hypocrella limushanensis*	YHH 2303016	-	OR828402	OR837108	-	OR832090	Wang et al. 2025
*Jenniferia cinerea*	BCC 06839	GQ249999	GQ249970	EU369048	EU369070	EU369009	Johnson et al. 2009; Mongkolsamrit et al. 2022
*Jenniferia cinerea*	BCC 2191	GQ250000	GQ249971	-	-	GQ250029	Mongkolsamrit et al. 2022
*Jenniferia cinerea*	NHJ 3510			EU369048	EU369070	EU369009	Johnson et al. 2009
*Jenniferia griseocinerea*	BCC 42062^T^	MZ684091	MZ684006	MZ707828	MZ707837	MZ707815	Mongkolsamrit et al. 2022
*Jenniferia thomisidarum*	BCC 37881^T^	MZ684099	MZ684010	MZ707830	MZ707843	MZ707823	Mongkolsamrit et al. 2022
*Kanoksria zaquensis*	HMAS 246915^T^	MT789699	MT789697	MT797810	-	MT797812	Wang et al. 2023
*Kanoksria zaquensis*	HMAS 246917	MT789698	MT789696	MT797809	-	MT797811	Wang et al. 2023
*Keithomyces carneus*	CBS 239.32	AY624171	EF468843	EF468894	EF468938	EF468789	Sung et al. 2007
*Keithomyces carneus*	CBS 399.59	MT078887	EF468842	EF468895	EF468939	EF468788	Sung et al. 2007
*Lecanicillium fusisporum*	CBS 164.70^T^	MH859538	AF339549	EF468889	-	EF468783	Sung et al. 2007; Zare and Gams 2001
*Lecanicillium lecanii*	CBS 101247	JN049836	AF339555	DQ522407	DQ522466	DQ522359	Spatafora et al. 2007; Zare and Gams 2001
*Lecanicillium lecanii*	CBS 102067^T^	MH862778	KM283795	KM283838	KM283860	KM283818	Vu et al. 2019; Sung et al. 2007
*Lecanicillium longisporum*	CBS 126.27^T^	OP756342	AF339556	KR064300	KR064303	KM283820	Vu et al. 2019; Sung et al. 2007
*Lecanicillium longisporum*	CBS 102072	-	KM283796	KM283839	KM283861	KM283819	Vu et al. 2019
*Leptobacillium chinense*	CGMCC 3.14970^T^	NR_155782	NG_069101	-	-	-	Liu and Cai 2012
*Leptobacillium leptobactrum*	CBS 774.69^T^	NR_154111	NG_069745	-	-	-	Vu et al. 2019
*Liangia sinensis*	YFCC 3103^T^	MN576831	MN576782	MN576842	MN576898	MN576952	Wang et al. 2020
*Liangia sinensis*	YFCC 3104	MN576832	MN576783	MN576843	MN576899	MN576953	Wang et al. 2020
*Marquandomyces marquandii*	CBS 182.27	MH854923	MH866418	EF468899	EF468942	EF468793	Vu et al. 2019; Sung et al. 2007
*Marquandomyces marquandii*	CBS 128893	MH865143	MH876582	-	-	-	Vu et al. 2019
*Matutinistella brasiliensis*	COAD 3683	PP836716	PP836706	PP855567	PP855539	PP855547	Custódio and Pereira 2025
*Matutinistella brasiliensis*	COAD 3686^T^	PP836714	PP836707	PP855576	PP855536	PP855559	Custódio and Pereira 2025
*Metacordyceps chlamydosporia*	JCM 18608	-	-	AB758684	AB758711	AB758481	Nonaka et al. 2013
*Metacordyceps chlamydosporia*	JCM 18603	-	-	AB758667	AB758694	AB758464	Nonaka et al. 2013
*Metarhizium anisopliae*	CBS 130.71^T^	MT078884	MT078853	-	-	MT078845	Mongkolsamrit et al. 2020
*Metarhizium flavoviride*	CBS 218.56^T^	MH857590	MH869139	-	-	KJ398787	Vu et al. 2019; Kepler et al. 2014
*Metarhiziopsis microspora*	CEHS133a	EF464589	EF464571	-	-	-	Li et al. 2008
*Metarhiziopsis microspora*	INEHS133a	EF464583	EF464572	-	-	-	Li et al. 2008
*Metapochonia bulbillosa*	CBS 145.70	MH859529	AF339542	-	-	EF468796	Vu et al. 2019; Sung et al. 2001, 2007
*Metapochonia cordycipiticonsociata*	CGMCC:317366^T^	KM263567	KM263574	KM263578	KM263580	KM263582	Huang et al. 2015
*Metapochonia goniodes*	CBS 891.72^T^	NR_154141	AF339550	KJ398642	DQ522458	DQ522354	Sung et al. 2001; Spatafora et al. 2007
*Metapochonia hahajimaensis*	JCM 34019^T^	LC529951	LC529959	-	-	LC529963	Kondo et al. 2020
*Metapochonia lutea*	BiMM-F96	MF983717	-	-	-	MF983718	Labuda et al. 2018
*Metapochonia microbactrospora*	CBS 101433^T^	NR_154140	MH874343	KJ398653	-	KJ398794	Vu et al. 2019; Kepler et al. 2014
*Metapochonia parasitica*	CBS 300.83^T^	MH861597	MH873320	-	-	-	Vu et al. 2019
*Metapochonia rubescens*	CBS 464.88^T^	MH862138	MH873830	EF468903	-	EF468797	Vu et al. 2019; Sung et al. 2007
*Metapochonia simonovicovae*	BiMM-F299^T^	PP085431	PP085432	-	-	PP085550	Crous et al. 2024
*Metapochonia suchlasporia*	CBS 251.83^T^	MH861580	MH873311	KJ398649	-	KJ398790	Vu et al. 2019; Kepler et al. 2014
*Metapochonia variabilis*	CGMCC:3.17925^T^	KU746684	KU746730	KY883214	KY883238	KX855229	Zhang et al. 2017
*Microhilum oncoperae*	ARSEF 4358	-	AF339532	EF468891	EF468936	EF468785	Sung et al. 2001, 2007
*Moelleriella alba*	BCC49409^T^	-	-	-	-	KX254423	Mongkolsamrit et al. 2015
*Moelleriella alba*	BCC49492	-	-	-	-	KX254424	Mongkolsamrit et al. 2015
*Mycophilomyces periconiae*	CPC 27558	KY173418	KY173509	-	-	KY173595	Crous et al. 2013
*Myriogenospora atramentosa*	AEG 96-32	-	AY489733	AY489665	-	AY489628	Castlebury et al. 2004
*Neoaraneomyces araneicola*	DY101711	MW730520	MW730609	-	MW753026	MW753033	Chen et al. 2022
*Neoaraneomyces araneicola*	DY101712	MW730522	MW730610	-	MW753027	MW753034	Chen et al. 2022
*Neobarya parasitica*	Marson s/n	KP899626	KP899626	-	-	-	Lawrey et al. 2015
*Neobaryopsis andensis*	A.F.25967-2^T^	NR_169713	NG_068701	-	-	-	Flakus et al. 2019
*Neobaryopsis andensis*	A.F.25967-1	MT153956	MT153985	-	-	-	Flakus et al. 2019
*Neohyperdermium piperis*	CBS 116719^T^	-	AY466442	DQ127240	EU369083	DQ118749	Chaverri et al. 2005; Bischoff and White Jr 2004
*Neohyperdermium pulvinatum*	P.C. 602	-	DQ118738	DQ127237	-	DQ118746	Bischoff and White Jr 2004
*Neolecanicillium sinensis*	DY091351^T^	PV082743	PV082860	-	-	PV171263	Chen et al. 2025
*Neolecanicillium sinensis*	DY091352	PV082744	PV082861	-	-	PV171264	Chen et al. 2025
*Neotorrubiella chinghridicola*	BCC 80733^T^	NR_175629	MK632097	MK632176	MK632149	MK632071	Thanakitpipattana et al. 2020
*Neotorrubiella chinghridicola*	BCC 39684	MK632038	MK632096	MK632181	-	MK632072	Thanakitpipattana et al. 2020
*Nigelia aurantiaca*	BCC13019	-	GU979948	-	GU979971	GU979957	Luangsa-ard et al. 2017
*Nigelia martiale*	EFCC 6863	-	JF415974	-	-	JF416016	Kepler et al. 2012a
*Nigrocornus scleroticus*	LB2015 09-18/3	-	MK660215	-	-	MN104682	Walker 2004
*Niveomyces coronatus*	NY04434800^T^	-	ON493606	ON513399	ON513400	ON513397	Araújo et al. 2022
*Niveomyces coronatus*	Niveo	-	ON493605	-	-	-	Araújo et al. 2022
*Niveomyces formicidarum*	BCC 83026^T^	ON103036	ON103161	ON286879	-	ON125019	Araújo et al. 2022
*Niveomyces hirsutellae*	BCC 36631^T^	ON103039	ON103164	ON286882	ON125033	ON125022	Araújo et al. 2022
*Niveomyces multisynnematus*	BCC 90308^T^	ON103038	ON103163	ON286881	ON125032	ON125021	Araújo et al. 2022
*Nuciformispora sinense*	DY05281^T^	PV082747	PV082864	PV171139	-	PV171267	Chen et al. 2025
*Nuciformispora sinense*	DY05282	PV082748	PV082865	PV171140	-	PV171268	Chen et al. 2025
*Ophiocordyceps arborescens*	NBRC 105891^T^	NR_147510	AB968414	-	AB968534	AB968572	Ban et al. 2015
*Ophiocordyceps brunneanigra*	TBRC 8093^T^	-	MF614654	MF614668	MF614681	MF614638	Luangsa-ard et al. 2018
*Ophiocordyceps unituberculata*	YFCC HU1301^T^	KY923212	KY923212	KY923218	KY923220	KY923216	Wang et al. 2018
*Ophiocordyceps xuefengensis*	GZUH2012HN14^T^	KC631802	-	KC631798	-	KC631793	Wen et al. 2013
*Orbiocrella petchii*	NHJ 5318	-	EU369040	EU369062	EU369080	EU369021	Johnson et al. 2009
*Orbiocrella petchii*	NHJ 6209	-	EU369039	EU369061	EU369081	EU369023	Johnson et al. 2009
*Papiliomyces albastromata*	YHH 2307002^T^	OR770519	OR770504	PP203269	PP479841	PP479838	Chen et al. 2024
*Papiliomyces albastromata*	YHH 2307003	OR770518	OR770503	PP203268	PP479840	PP479837	Chen et al. 2024
*Paracorniculantispora sinensis*	ZY06251^T^	PV082753	PV082870	PV171145	PV171195	PV171273	Chen et al. 2025
*Paracorniculantispora sinensis*	ZY06252	PV082754	PV082871	PV171146	PV171196	PV171274	Chen et al. 2025
*Parahevansia koratensis*	BCC01485	GQ250010	GQ249981	-	-	GQ250031	Mongkolsamrit et al. 2022
*Parahevansia websteri*	NHJ 2662	GQ250008	GQ249982	-	-	GQ250032	Mongkolsamrit et al. 2022
*Paraisaria gracilioides*	HUA 186095	-	-	KP212914	-	KM411994	Sanjuan et al. 2015
*Paraisaria gracilis*	EFCC 3101	-	EF468810	EF468858	EF468913	EF468750	Sung et al. 2007
*Paraisaria heteropoda*	EFCC 10125	JN049852	EF468812	EF468860	EF468914	EF468752	Sung et al. 2007
*Paraisaria heteropoda*	OSC 106404	-	AY489722	AY489651	-	AY489617	Castlebury et al. 2004
*Paraisaria orthopterorum*	BBC 88305	MH754742	MK332583	MK214084	-	MK214080	Mongkolsamrit et al. 2019
*Paraisaria phuwiangensis*	BBH 43492	MH188541	MH201169	MH211352	-	MH211355	Mongkolsamrit et al. 2019
*Paraisaria rosea*	HKAS102546	MN947222	MN943842	MN929081	MN929084	MN929088	Wei et al. 2021
*Paraisaria yodhathaii*	BBH43163	MH188539	MK332584	MH211349	-	MH211353	Mongkolsamrit et al. 2019
*Parengyodontium album*	CBS 836.71	LC092882	MH872118	-	-	LC382178	Tsang et al. 2016; Vu et al. 2019
*Parengyodontium torokii*	FJII-L10-SW-P1^T^	MT704894	-	-	-	-	Parker et al. 2022
*Parengyodontium torokii*	CBS 368.72	MH860502	MH872217	-	-	LC382183	Vu et al. 2019; Tsang et al. 2016
*Parametarhizium changbaiense*	CGMCC 19143	MN589741	MN589994	-	MT921829	MN908589	Gao et al. 2021
*Parametarhizium hingganense*	CGMCC 19144	MN055703	MN061635	-	MT939494	MN065770	Gao et al. 2021
*Paramoelleriella curvospora*	YHH 2305001	-	-	OR837111	-	OR854262	Wang et al. 2025
*Paramoelleriella curvospora*	YHH 2305002	-	OR835994	OR837112	-	OR854263	Wang et al. 2025
*Paraneoaraneomyces sinensis*	ZY 22.006	OQ709254	OQ709260	-	-	-	Zhang et al. 2023
*Paraneoaraneomyces sinensis*	ZY 22.007	OQ709255	OQ709261	-	-	-	Zhang et al. 2023
*Paraneoaraneomyces sinensis*	ZY 22.008	OQ709256	OQ709262	-	-	-	Zhang et al. 2023
*Perennicordyceps prolifica*	NBRC 101750	JN943340	JN941433	JN992442	AB972957	AB972953	Schoch et al. 2012
*Perennicordyceps prolifica*	TNS-F-18547	KF049660	KF049632	KF049649	KF049670	KF049687	Kepler et al. 2013
*Perennicordyceps prolifica*	TNS-F-18481	KF049659	KF049631	KF049648	-	KF049686	Kepler et al. 2013
*Periglandula ipomoeae*	IasaF13	-	-	-	KP689517	KP689568	Kepler et al. 2014
*Petchia siamensis*	BCC68421	MK632050	MK632088	MK632164	MK632141	MK632059	Thanakitpipattana et al. 2020
*Petchia siamensis*	BCC73636^T^	MK632051	MK632089	-	MK632138	MK632060	Thanakitpipattana et al. 2020
*Pleurocordyceps sinensis*	CN 80-2^T^	HQ832884	HQ832886	HQ832888	HQ832889	HQ832890	Wang et al. 2021
*Pleurocordyceps sinensis*	MFLU 21-0269	OQ172080	OQ172050	OQ459768	OQ459814	OQ459742	Xiao et al. 2023
*Pleurodesmospora coccorum*	CBS 460.73	MH860743	MH872455	-	-	-	Vu et al. 2019
*Pleurodesmospora lepidopterorum*	DY 10501^T^	MW826576	-	MW834315	MW834316	MW834317	Chen et al. 2021b
*Pleurodesmospora lepidopterorum*	DY 10502	MW826577	-	-	MW834318	MW834319	Chen et al. 2021b
*Pochonia sinensis*	ZY 22.009	OQ709257	OQ709263	-	-	-	Zhang et al. 2023
*Pochonia sinensis*	ZY 22.010	OQ709258	OQ709264	-	-	-	Zhang et al. 2023
*Polycephalomyces formosus*	NBRC 109993^T^	MN586833	MN586842	MN598048	MN598064	MN598057	Wang et al. 2021
*Polycephalomyces formosus*	NBRC 109994	MN586834	MN586843	MN598049	MN598065	MN598058	Wang et al. 2021
*Polymicrospora caiyangheensis*	YHH 2309001^T^	-	OR835995	OR837113	-	OR854247	Wang et al. 2025
*Polymicrospora caiyangheensis*	YHH 2309002	-	OR835996	OR837114	-	OR854248	Wang et al. 2025
*Polystromomyces araneae*	BCC 93301^T^	MZ684101	MZ684016	MZ707832	MZ707845	MZ707825	Mongkolsamrit et al. 2022
*Pseudogibellula formicarum*	BCC 84257	MT508782	MT512653	-	-	MT533480	Mongkolsamrit et al. 2021
*Pseudogibellula formicarum*	CBS 433.73	MH860731	MH872442	MT533475	-	MT533481	Vu et al. 2019; Mongkolsamrit et al. 2021
*Pseudolecanicillium caatingaense*	URM8446^T^	ON862933	ON862925	-	OP290514	OP290526	Alves et al. 2022
*Pseudolecanicillium caatingaense*	URM8442	ON862934	ON862926	-	OP290513	OP290525	Alves et al. 2022
*Pseudometarhizium araneogenum*	DY101741	MW730532	MW730618	-	MW753030	MW753037	Chen et al. 2022
*Pseudometarhizium araneogenum*	DY101801^T^	MW730536	MW730623	-	MW753032	MW753039	Chen et al. 2022
*Pseudoniveomyces arachnovorum*	BCC 95818^T^	OR098526	-	OR133173	OR133174	OR133172	Kobmoo et al. 2023
*Pseudoniveomyces blattae*	BCC 53567^T^	ON103042	ON103167	ON286885	ON125036	-	Kobmoo et al. 2023
*Pseudoniveomyces blattae*	BCC 53568	ON103043	ON103168	ON286886	ON125037	ON125025	Kobmoo et al. 2023
*Purpureocillium atypicolum*	CBS 744.73	-	EF468841	EF468892	-	EF468786	Sung et al. 2007
*Purpureocillium atypicolum*	OSC 151901	-	KJ878880	KJ878994	-	KJ878961	Quandt et al. 2014
*Purpureocillium lavendulum*	CBS 128677^T^	MH864976	FR775489	FR775512	FR775538	FR775516	Perdomo et al. 2013
*Purpureocillium lilacinum*	CBS 431.87	AY624188	EF468844	EF468897	EF468940	EF468791	Sung et al. 2007
*Purpureocillium lilacinum*	CBS 284.36^T^	AY624189	FR775484	EF468898	EF468941	EF468792	Sung et al. 2007
*Purpureomyces khaovaiensis*	BCC1376	KX983460	KX983462	-	KX983465	KX983457	Luangsa-ard et al. 2017b
*Purpureomyces khaovaiensis*	BCC 88441	MN781916	MN781877	-	MN781824	MN781734	Mongkolsamrit et al. 2020
*Regiocrella camerunensis*	ARSEF 7682	-	DQ118735	DQ127234	-	DQ118743	Chaverri et al. 2005
*Regiocrella sinensis*	CUP-CH 2640	-	DQ118736	DQ127235	-	DQ118744	Chaverri et al. 2005
*Rotiferophthora angustispora*	CBS 101437	OR600291	AF339535	DQ522402	DQ522460	PP407582	Sung et al. 2001; Spatafora et al. 2007
*Rubrumomyces sinensis*	ZY06111^T^	PV082757	PV082874	PV171147	-	PV171275	Chen et al. 2025
*Rubrumomyces sinensis*	ZY06112	PV082758	PV082875	PV171148	-	PV171276	Chen et al. 2025
*Samsoniella inthanonensis*	TBRC 7915^T^	NR_164420	NG_069500	MF140790	MF140815	MF140849	Mongkolsamrit et al. 2018
*Samsoniella inthanonensis*	TBRC 7916	MF140760	MF140724	MF140789	MF140814	MF140848	Mongkolsamrit et al. 2018
*Samuelsia chalalensis*	P.C. 560	-	EU392637	EU392743	-	EU392691	Chaverri et al. 2008
*Samuelsia rufobrunnea*	P.C.613	-	AY986918	DQ000345	-	AY986944	Chaverri et al. 2005
*Shimizuomyces paradoxus*	EFCC 6279	JN049847	EF469084	EF469100	EF469117	EF469071	Sung et al. 2007
*Shimizuomyces paradoxus*	EFCC 6564	-	EF469083	EF469101	EF469118	EF469072	Sung et al. 2007
*Simplicillium lanosoniveum*	CBS 704.86	AJ292396	AF339553	DQ522406	-	DQ522358	Zare et al. 2000; Sung et al. 2001; Spatafora et al. 2007
*Simplicillium lanosoniveum*	CBS 123.42^T^	NR_171734	NG_068571	-	-	-	Zare et al. 2000
*Subuliphorum camptosporum*	CBS:756.69	OQ429878	OQ430129	-	OQ454278	-	Hou et al. 2023
*Subuliphorum camptosporum*	CBS:835.91	-	OQ430128	-	-	OQ471209	Hou et al. 2023
*Sungia yongmunensis*	EFCC2131	NR169650	NG075160	-	-	-	Mongkolsamrit et al. 2020
*Tolypocladium album*	CBS 869.73^T^	AB457005	-	-	-	-	Quandt et al. 2014
*Tolypocladium album*	MS 490	JX155907	-	-	-	-	Quandt et al. 2014
*Tolypocladium amazonense*	CBS 136895^T^	JQ905653	KF747134	KF747214	-	KF747099	Gazis et al. 2014
*Tolypocladium bacillisporum*	C23	LC684522	LC684522	-	-	LC684525	Yamamoto et al. 2022
*Tolypocladium capitatum*	NBRC 100997	JN943313	JN941401	JN992474	AB968558	AB968597	Schoch et al. 2012
*Tolypocladium capitatum*	NBRC 106325	JN943314	JN941402	JN992473	AB968559	AB968598	Schoch et al. 2012
*Tolypocladium cicadae*	YFCC 8003	-	MT184208	MT184198	MT184188	MN960689	Yu et al. 2021
*Tolypocladium cicadae*	YFCC 5062	-	MT184206	MT184196	MT184186	MN969443	Yu et al. 2021
*Tolypocladium cucullae*	GZU A-77	MW798789	MW798787	-	-	-	Wijayawardene et al. 2021
*Tolypocladium cucullae*	HKAS 55588^T^	MW798788	MW798786	-	-	-	Wijayawardene et al. 2021
*Tolypocladium cylindrosporum*	ARSEF 2920^T^	-	MH871712	MG228384	MG228387	MG228390	Vu et al. 2019;
*Tolypocladium cylindrosporum*	YFCC 1805001	MK984581	MK984577	MK984584	MK984573	MK984569	Wang et al. 2022
*Tolypocladium dujiaolongae*	ZBAH632	KF696557	-	-	-	-	Li et al. 2018
*Tolypocladium dujiaolongae*	RCEF6201	KF696558	-	-	-	-	Li et al. 2018
*Tolypocladium endophyticum*	MS337	JQ905657	KF747136	KF747215	-	KF747101	Gazis et al. 2014
*Tolypocladium endophyticum*	MX486	KF747245	KF747152	KF747232	-	KF747116	Gazis et al. 2014
*Tolypocladium extinguens*	CBS 345.77^T^	MH861072	MH872842	-	-	-	Vu et al. 2019
*Tolypocladium flavonigrum*	BCC 66576	-	MN337287	-	-	MN338495	Crous et al. 2020
*Tolypocladium flavonigrum*	BCC 66580	-	MN337289	MN338494	-	MN338497	Crous et al. 2020
*Tolypocladium fractum*	OSC 110990	-	DQ518759	DQ522373	DQ522425	DQ522328	Spatafora et al. 2007
*Tolypocladium fumosum*	CBS H-22968^T^	KU925171	KU985053	-	-	-	Crous et al. 2017
*Tolypocladium geodes*	CBS 126054	MH864065	MH875520	-	-	-	Vu et al. 2019
*Tolypocladium geodes*	CBS 723.70^T^	MH859919	-	-	-	-	Vu et al. 2019
*Tolypocladium globosum*	KNUF-22-14A^T^	LC731698	LC731699	-	-	-	Das et al. 2023
*Tolypocladium globosum*	KNUF-22-15A	LC731703	LC731704	-	-	-	Das et al. 2023
*Tolypocladium guangdongense*	GDGM 24020^T^	EU039881	-	-	-	-	Lin et al. 2008
***Tolypocladium guizhouense***	**TR05051^T^**	**PZ263738**	**PZ263748**	**PZ277170**	**PZ277176**	**PZ277182**	**This study**
***Tolypocladium guizhouense***	**TR05052**	**PZ263739**	**PZ263749**	**PZ277171**	**PZ277177**	**PZ277183**	**This study**
*Tolypocladium inegoense*	SU-15	-	DQ118741	DQ127243	-	DQ118752	Chaverri et al. 2005
*Tolypocladium inflatum*	OSC 71235	JN049844	EF469077	EF469090	EF469108	EF469061	Sung et al. 2007
*Tolypocladium inusitaticapitatum*	HKAS 112152	MW537735	MW537718	-	MW507529	MW507527	Yu et al. 2021
*Tolypocladium inusitaticapitatum*	HKAS 112153	-	MW537719	-	MW507530	MW507528	Yu et al. 2021
*Tolypocladium japonicum*	NBRC 9647	-	OP207732	OP223124	OP223134	OP223146	Dong et al. 2022
*Tolypocladium jezoense*	NBRC 106328	-	OP207733	OP223125	OP223135	OP223147	Dong et al. 2022
*Tolypocladium lignicola*	CBS 489.83^T^	MH861631	MH873346	-	-	-	Vu et al. 2019
*Tolypocladium longisegmentatum*	OSC 110992	-	EF468816	EF468864	EF468919	-	Sung et al. 2007
*Tolypocladium nubicola*	CBS 568.84^T^	MH861780	MH873478	-	-	-	Vu et al. 2019
*Tolypocladium ophioglossoides*	NBRC 100998	JN943319	JN941406	JN992469	AB968563	AB968602	Sung et al. 2007; Ban et al. 2015
*Tolypocladium ophioglossoides*	NBRC 106330	JN943321	JN941407	JN992468	AB968564	AB968603	Sung et al. 2007; Ban et al. 2015
*Tolypocladium ovalisporum*	CBS 700.92^T^	AB457006	-	-	-	-	Quandt et al. 2014
*Tolypocladium paradoxum*	NBRC 100945	JN943323	JN941410	JN992465	AB968560	AB968599	Sung et al. 2007; Ban et al. 2015
*Tolypocladium paradoxum*	YFCC 882	-	OP207734	OP223126	OP223136	OP223148	Dong et al. 2022
*Tolypocladium pennsylvanicum*	CBS 152275^T^	NR_199123	NG_244381	-	-	-	Crous et al. 2024
*Tolypocladium pennsylvanicum*	CPC 45907	PQ498950	PQ498999	PQ497759	-	PQ497746	Crous et al. 2024
*Tolypocladium phycosomatis*	YFCC 24099493^T^	PQ864791	PQ864789	PV017453	-	PV017452	Li et al. 2025
*Tolypocladium pseudoalbum*	YFCC 875^T^	-	OP207737	OP223129	OP223139	OP223151	Dong et al. 2022
*Tolypocladium pseudoalbum*	YFCC 876	-	OP207738	OP223130	OP223140	OP223152	Dong et al. 2022
*Tolypocladium pustulatum*	MRL GB6597	AF389189	AF389190	-	-	-	Bills et al. 2002
*Tolypocladium pustulatum*	MRL MF5368LR	-	AF373282	-	-	-	Bills et al. 2002
*Tolypocladium queenslandicum*	BRIP 72623a	NR_189998	OR452936	-	OR459911	OR459922	Tan and Shivas 2023
*Tolypocladium reniformisporum*	YFCC 1805002^T^	MK984582	MK984578	MK984585	MK984574	MK984570	Wang et al. 2022
*Tolypocladium sinense*	CS7	KX082969	-	-	-	-	Das et al. 2023
*Tolypocladium sobaeksanense*	KNUF-22-014	LC802118	LC802123	LC802125	-	LC802124	Song et al. 2024
*Tolypocladium subparadoxum*	NBRC 106958	-	OP207735	OP223127	OP223137	OP223149	Dong et al. 2022
*Tolypocladium subparadoxum*	YFCC 879^T^	-	OP207736	OP223128	OP223138	OP223150	Dong et al. 2022
*Tolypocladium subtropicale*	JMS200^T^	ON490898	ON495714	ON512625	-	ON512593	Soares et al. 2023
*Tolypocladium terrae*	KNUF-23-321C^T^	PQ773315	PQ773314	-	-	PQ772836	Lim et al. 2025
***Tolypocladium tongrenense***	**TR05201^T^**	**PZ263740**	**PZ263750**	**PZ277172**	**PZ277178**	**PZ277184**	**This study**
***Tolypocladium tongrenense***	**TR05202**	**PZ263741**	**PZ263751**	**PZ277173**	**PZ277179**	**PZ277185**	**This study**
*Tolypocladium trecense*	JMS111^T^	ON490895	ON495712	ON512645	-	ON512590	Soares et al. 2023
*Tolypocladium tropicale*	CBS 136897^T^	KF747254	KF747125	KF747204	-	KF747090	Gazis et al. 2014
*Tolypocladium tropicale*	MX338	KF747259	KF747149	KF747229	-	KF747113	Gazis et al. 2014
*Tolypocladium tundrense*	CBS 569.84^T^	MH861781	MH873479	-	-	-	Vu et al. 2019
*Tolypocladium valdiviae*	LSB131^T^	OP345930	OP345928	-	-	OP352233	Gallardo-Pillancari et al. 2023
*Tolypocladium valdiviae*	LSB132	OP345931	OP345929	-	-	-	Gallardo-Pillancari et al. 2023
*Tolypocladium valliforme*	DAOM 196368	AY245640	-	-	-	-	Quandt et al. 2014
*Tolypocladium varium*	CBS 429.94^T^	MH862472	-	-	-	-	Vu et al. 2019
*Tolypocladium yunnanense*	YFCC 877^T^	-	OP207739	OP223131	-	OP223153	Dong et al. 2022
*Tolypocladium yunnanense*	YFCC 878	-	OP207740	OP223132	-	OP223154	Dong et al. 2022
***Tolypocladium zongqii***	**TR05301^T^**	**PZ263742**	**PZ263752**	**PZ277174**	**PZ277180**	**PZ277186**	**This study**
***Tolypocladium zongqii***	**TR05302**	**PZ263743**	**PZ263753**	**PZ277175**	**PZ277181**	**PZ277187**	**This study**
*Torrubiellomyces zombiae*	NY04434801^T^	-	ON493602	ON513398	ON513402	ON513396	Araújo et al. 2022
*Torrubiellomyces zombiae*	FieldB	-	ON493603	-	-	ON513395	Araújo et al. 2022
*Torrubiellomyces sessilis*	CBM FA-39236^T^	-	AB721304	-	-	-	Kaitsu et al. 2013
*Tyrannicordyceps fratricida*	TNSF19011	JQ349068	JQ257023	-	JQ257021	JQ257028	Kepler et al. 2012b
*Ustilaginoidea virens*	ATCC 16180	-	-	-	JQ257019	JQ257026	Kepler et al. 2012b
*Verticillium epiphytum*	CBS 384.81	MH861358	MH873113	DQ522409	DQ522469	DQ522361	Vu et al. 2019; Spatafora et al. 2007
*Yosiokobayasia kusanagiensis*	TNS F18494	JN049873	JF415972	JN049890	-	JF416014	Kepler et al. 2012a
*Zarea fungicola*	CBS 992.69^T^	NR_119653	KM283792	-	KM283857	KM283816	Vu et al. 2019
*Zongqiomyces araneicola*	ZY09191^T^	PV082797	PV082914	-	-	PV171315	Chen et al. 2025
*Zongqiomyces araneicola*	ZY09192	PV082898	PV082915	-	-	PV171316	Chen et al. 2025
*Zouia cauligalbarum*	GZUIFRZHJ01^T^	MH730663	MH730667	MH801922	MH801924	MH801920	Zhou et al. 2018
*Zouia cauligalbarum*	GZUIFRZHJ02	MH730664	MH730668	MH801923	MH801925	MH801921	Zhou et al. 2018

Note. Newly generated species and their strain numbers are in bold. Ex-type strains are denoted by superscript T and unavailable sequences were indicated by hyphen “-”.

### Sequence alignments and Phylogenetic analyses

In this study, DNA sequences were edited using DNASTAR™ Lasergene version 6.0. Sequences of ITS, LSU, *rpb1*, *rpb2* and *tef*-1α for *Tolypocladium* species and new strains were obtained from GenBank following Yu et al. (2021), Dong et al. (2022), Chen et al. (2025) and Li et al. (2025), with additional sequences selected through BLASTn searches. MAFFT v.7.037b (Katoh and Standley 2013) and MEGA6 (Tamura et al. 2013) were used to align and edit all the sequences. SequenceMatrix v.1.7.8 was used to obtain combined sequences for the analysis (ITS, LSU, *rpb1*, *rpb2*, *tef*-1α) (Vaidya et al. 2011). ModelFinder (Kalyaanamoorthy et al. 2017), implemented in PhyloSuite v. 1.2.2 (Zhang et al. 2020), was used to select the best-fit substitution models for the phylogenetic analyses.

Bayesian inference (BI) and maximum likelihood (ML) analyses were performed independently on the combined sequence dataset. For BI analysis, the combined dataset was partitioned according to the five loci, and the GTR+F+I+G4 model was applied to each partition. MrBayes v. 3.2 (Ronquist et al. 2012) was run through PhyloSuite, which was used only as a graphical interface for preparing and executing the MrBayes analysis. Two independent runs, each consisting of four concurrent Markov chains, were conducted for 10 million generations. Trees were sampled every 1,000 generations, and the analysis was terminated when the average standard deviation of split frequencies fell below 0.01. The first 25% of the sampled trees were discarded as burn-in, and the remaining trees were used to calculate Bayesian posterior probabilities (PP). Tracer v. 1.5 (Drummond and Rambaut 2007) was used to assess convergence and effective sample sizes (ESS), with ESS values exceeding 200 indicating adequate sampling. Maximum likelihood (ML) analysis was performed independently using IQ-TREE v. 2.0 (Trifinopoulos et al. 2016). The TN+R2+F substitution model was selected as the best-fit model for the ML analysis. Branch support was assessed using the ultrafast bootstrap (UFBoot) method with 10,000 replicates. SH-aLRT support values were also calculated with 1,000 replicates during the IQ-TREE analysis but were not used for the node labels presented in the final phylogenetic tree. In the resulting phylogenetic tree, node values are presented as UFBoot support values/Bayesian posterior probabilities (PP).

### Genealogical Concordance Phylogenetic Species Recognition (GCPSR) analysis

The Genealogical Concordance Phylogenetic Species Recognition model was applied to analyze the related species. The pairwise homoplasy index (PHI) (Bruen et al. 2006) is a model test based on the observation that multiple gene phylogenies are concordant among species and discordant within species due to recombination and mutations. The test was performed in SplitsTree4 (Huson and Bryant 2006) as described by Quaedvlieg et al. (2014) to determine the recombination level within phylogenetically closely related species using a five-locus concatenated dataset. The new species and their closely related species were analyzed using this model. The relationships between closely related species were visualized by constructing a split graph using both the LogDet transformation and the splits decomposition option.

## Results

### Phylogenetic analyses

To clarify the taxonomic position of *Tolypocladium* species and evaluate the phylogenetic placement of the new strains, phylogenetic analyses of *Tolypocladium* species and related taxa were conducted as part of the phylogenetic reassessment of the genus. *Calcarisporium cordycipiticola* Jing Z. Sun, C.H. Dong, Xing Z. Liu & K.D. Hyde (CGMCC 3.17904 and CGMCC 3.17905) was selected as the outgroup. The combined dataset comprised 202 taxa and 3,424 characters (ITS, 663; LSU, 708; *rpb1*, 562; *rpb2*, 715 and *tef*-1α, 776), including gaps. For maximum likelihood analysis, the TN+R2+F substitution model was identified as the best-fit model, yielding a best-scoring tree with a log-likelihood value of -103,961.131. The estimated nucleotide frequencies were A = 0.234, C = 0.286, G = 0.279, and T = 0.201. The two FreeRate categories had proportions and rates of 0.659 and 0.132, and 0.341 and 2.674, respectively.

**Figure 1.**
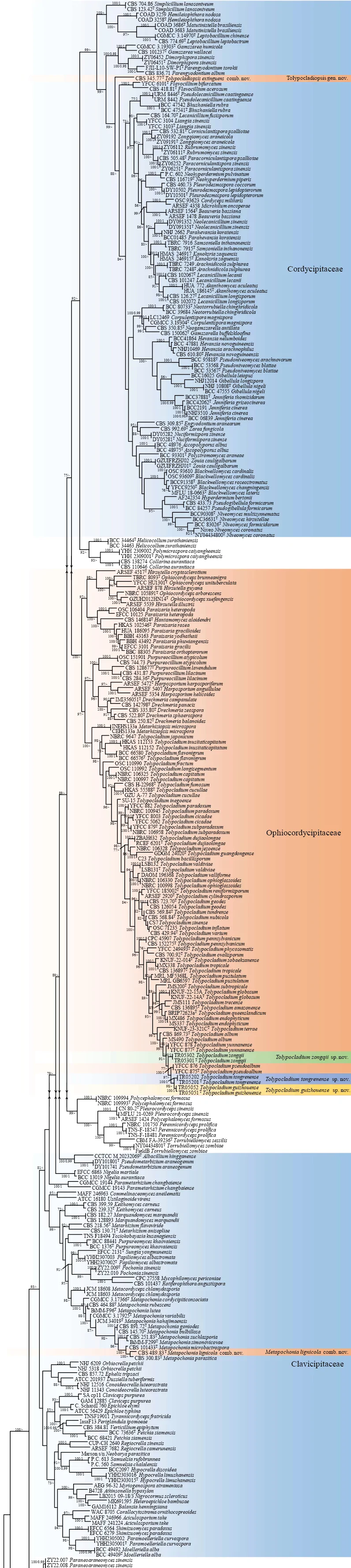
Phylogram retrieved from IQTREE of the new species and *Tolypocladium* species using the combined dataset of ITS, LSU, *rpb1*, *rpb2* and *tef*-1α gene regions. Node values are presented as UFBoot support values/Bayesian posterior probabilities (ML value above 70% and BI value above 0.95). The tree is rooted with *Calcarisporium cordycipiticola* (CGMCC 3.17904 and CGMCC 3.17905). Ex-types, new strains and new species are indicated by the superscript “T” and in bold, respectively.

Bayesian inference (BI) was conducted using partition-specific models, with GTR+F+I+G4 applied to the ITS, LSU, *rpb1*, *rpb2*, and *tef*-1α partitions. The ML and BI analyses produced largely congruent topologies (Fig. 1), with most nodes receiving strong statistical support. The majority of the sampled genera formed distinct monophyletic groups in the phylogenetic analyses.

### Clavicipitaceae

In the phylogenetic tree (Fig. 1), *Tolypocladium lignicola* G.L. Barron (CBS 489.83) was placed within the genus *Metapochonia* Kepler, S.A. Rehner & Humber.

### Cordycipitaceae

*Tolypocladium extinguens* (CBS 345.77) is placed within *Cordycipitaceae*, where it is phylogenetically positioned in close proximity to *Dimorphspora* Wan H. Chen, Y.F. Han & J.D. Liang, *Flavocillium* Hong Yu bis, Y.B. Wang, Yao Wan, Q. Fan & Zhu L. Yang, *Gamszarea* Z.F. Zhang & L. Cai, and *Parengyodontium* C.C.C. Tsang, J.F.W. Chan, W.M. Pong, J.H.K. Chen, A.H.Y. Ngan, M. Cheung, C.K.C. Lai, D.N.C. Tsang, S.K.P. Lau & P.C.Y. Woo, forming a distinct clade separate from these genera.

### Ophiocordycipitaceae

The new strains TR05051, TR05052, TR05201, TR05202, TR05301, and TR05302 formed three distinct, well-supported clades (100% ML/1 PP) in *Tolypocladium* s. str. These clades were further grouped with *Tolypocladium pseudoalbum*, with high statistical support (95% ML).

Additionally, *Metarhiziopsis microspora* D.W. Li, R.S. Cowles & Vossbr. (CEHS133a and INEHS133a) was grouped as a sister clade to *Tolypocladium* s. str.

### GCPSR analysis

**Figure 2.**
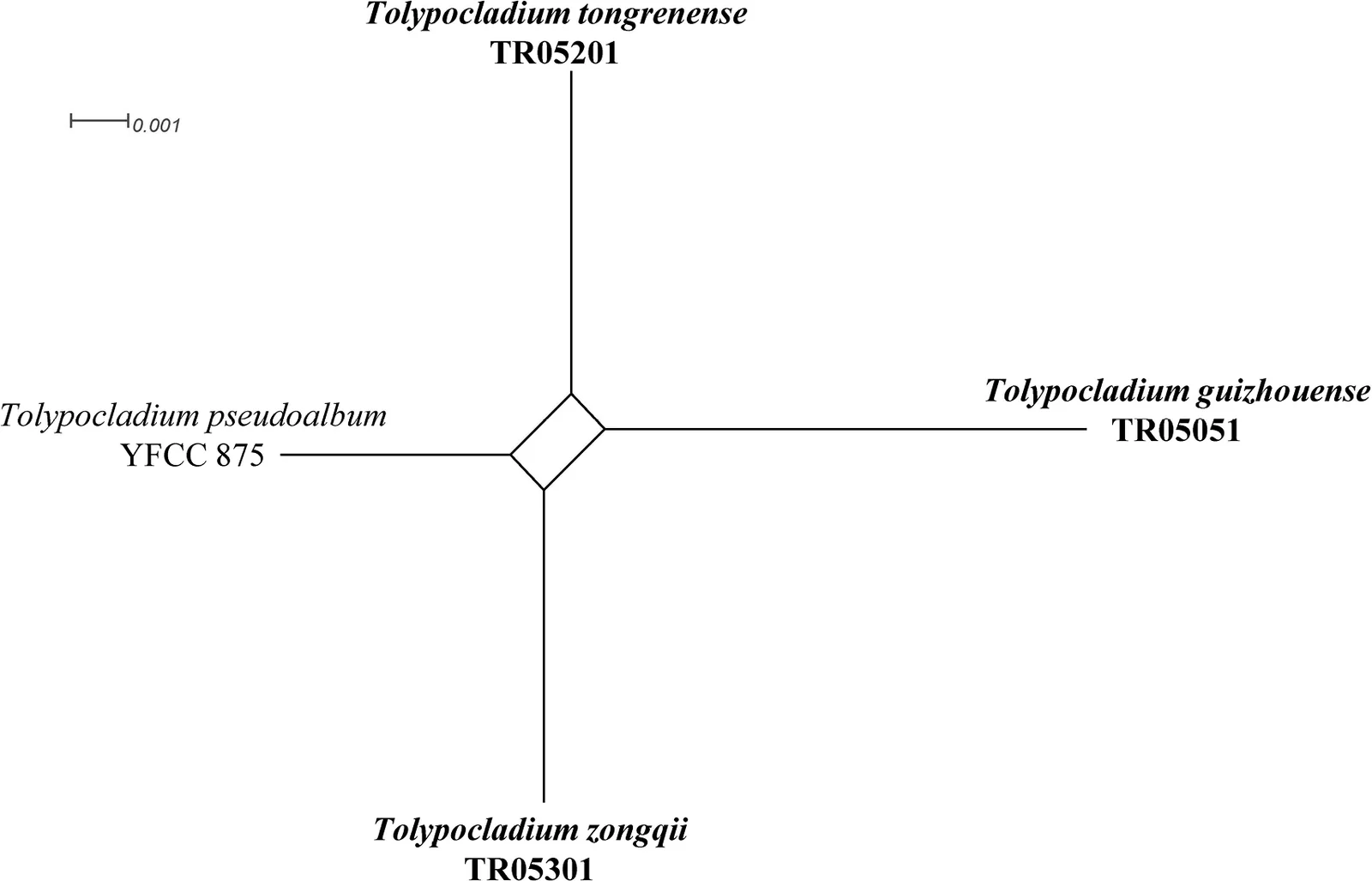
Results of the pairwise homoplasy index (PHI) test of the new strains and their closely-related species using both LogDet transformation and splits decomposition. PHI test results (Φ_w_) < 0.05 indicate significant recombination within the dataset. The new strains are in bold type.

A five-locus concatenated dataset (ITS, LSU, *rpb1*, *rpb2* and *tef*-1α) was used to determine the recombination level within *Tolypocladium guizhouense* (TR05051), *T. tongrenense* (TR05201), *T. zongqii* (TR05301), and *T. pseudoalbum* (YFCC 875). Chaiwan et al. (2022) noted that a PHI below the 0.05 threshold (Φ_w_ < 0.05) indicates significant recombination in the dataset. This means that related species in a group and recombination levels are not different. If the PHI exceeds the 0.05 threshold (Φ_w_ > 0.05), it indicates that it is not significant, which means that the related species at the group level are different. The result of the pairwise homoplasy index (PHI) test of *Tolypocladium guizhouense*, *T. tongrenense*, *T. zongqii*, and *T. pseudoalbum* was 0.05942 and revealed that those species were different (Fig. 2).

### Taxonomy

***Cordycipitaceae* Kreisel ex G.H. Sung, J.M. Sung, Hywel-Jones & Spatafora, Stud. Mycol. 57: 48 (2007)**

#### Tolypocladiopsis

Taxon classificationFungiSordariomycetesCordycipitaceae

W.H. Chen, Y.F. Han & J.D. Liang gen. nov.

640CD79A-F450-5994-B1AB-C83F1750108D

Index Fungorum: IF905330

##### Etymology.

Refers to the morphological similarity of this genus to *Tolypocladium*, while being phylogenetically distinct and placed within a different family (*Cordycipitaceae*).

##### Description.

Colonies on agar media grow very slowly, but sporulate abundantly within 7 days. Conidiophores bearing several regularly verticillate branches, which terminate in whorls of phialides. Phialides small, consisting of a swollen base and a thin neck, giving rise to conidia in slimy heads. Conidia are one-celled, ellipsoidal to subglobose, sometimes kidney-shaped.

##### Note.

Samson and Soares Jr (1984) introduced *Tolypocladium extinguens* from *Arachnocampa luminosa* in New Zealand. Molecular phylogenetic analysis based on the concatenated dataset (Fig. 1) revealed that *Tolypocladium extinguens* (CBS 345.77, ex-type) formed a distinct lineage within the clade of *Cordycipitaceae*, rather than *Ophiocordycipitaceae*, and showed a close relationship with the genera *Dimorphspora*, *Flavocillium*, *Gamszarea*, and *Parengyodontium*. Morphologically, *Tolypocladium extinguens* was distinguished from *Dimorphspora*, *Flavocillium*, *Gamszarea* and *Pseudoniveomyces* by its paecilomyces-like conidiogenous structure and ellipsoidal to subglobose, sometimes kidney-shaped conidia. The combined evidence from morphological characteristics and robust molecular phylogenetic analyses supports the establishment of a new genus, *Tolypocladiopsis*, to accommodate *Tolypocladium extinguens* within *Cordycipitaceae*.

##### Type species.

*Tolypocladiopsis extinguens* (Samson & Soares) W.H. Chen, F. Han & D. Liang.

#### Tolypocladiopsis extinguens

Taxon classificationFungiSordariomycetesCordycipitaceae

(Samson & Soares) W.H. Chen, Y.F. Han & J.D. Liang comb. nov.

31308FAB-EE05-5AD0-9ADE-80B73DF10089

Index Fungorum: IF905331

107332

Tolypocladium extinguens Samson & Soares, J. Invert. Path. 43(2): 133 (1984). Basionym.

##### Type.

New Zealand • Waitomo, Morin Cave, on *Arachnocampa luminosa*, 27 January, 1982, collected by D. Williams and P. Johnston, ex-type CBS 345.77, isolated by G.J. Samuels.

##### Description.

See Samson and Soares Jr (1984).

##### Note.

In our phylogenetic tree (Fig. 1), *Tolypocladium extinguens* (CBS 345.77) formed a monophyletic clade in *Cordycipitaceae* and has a close relationship with *Dimorphspora*, *Flavocillium*, *Gamszarea*, and *Parengyodontium*. However, *Tolypocladium extinguens* was significantly different from *Dimorphspora*, *Flavocillium*, *Gamszarea*, and *Parengyodontium* by its paecilomyces-like conidiogenous structure and ellipsoidal to subglobose, sometimes kidney-shaped conidia. Thus, the new genus *Tolypocladiopsis* is proposed, and the new combination species is named as *T. extinguens*.

### *Ophiocordycipitaceae* G.H. Sung, J.M. Sung, Hywel-Jones & Spatafora, Stud. Mycol. 57: 35 (2007)

***Tolypocladium* W. Gams, Persoonia 6(2): 185 (1971)**

#### Tolypocladium guizhouense

Taxon classificationFungiSordariomycetesOphiocordycipitaceae

W.H. Chen, Y.F. Han & J.D. Liang sp. nov.

3A6F1DEE-3246-5498-B74A-EABD10CC057D

Index Fungorum: IF905334

Fig. 3

##### Etymology.

Refers to the type locality, Guizhou Province.

**Figure 3.**
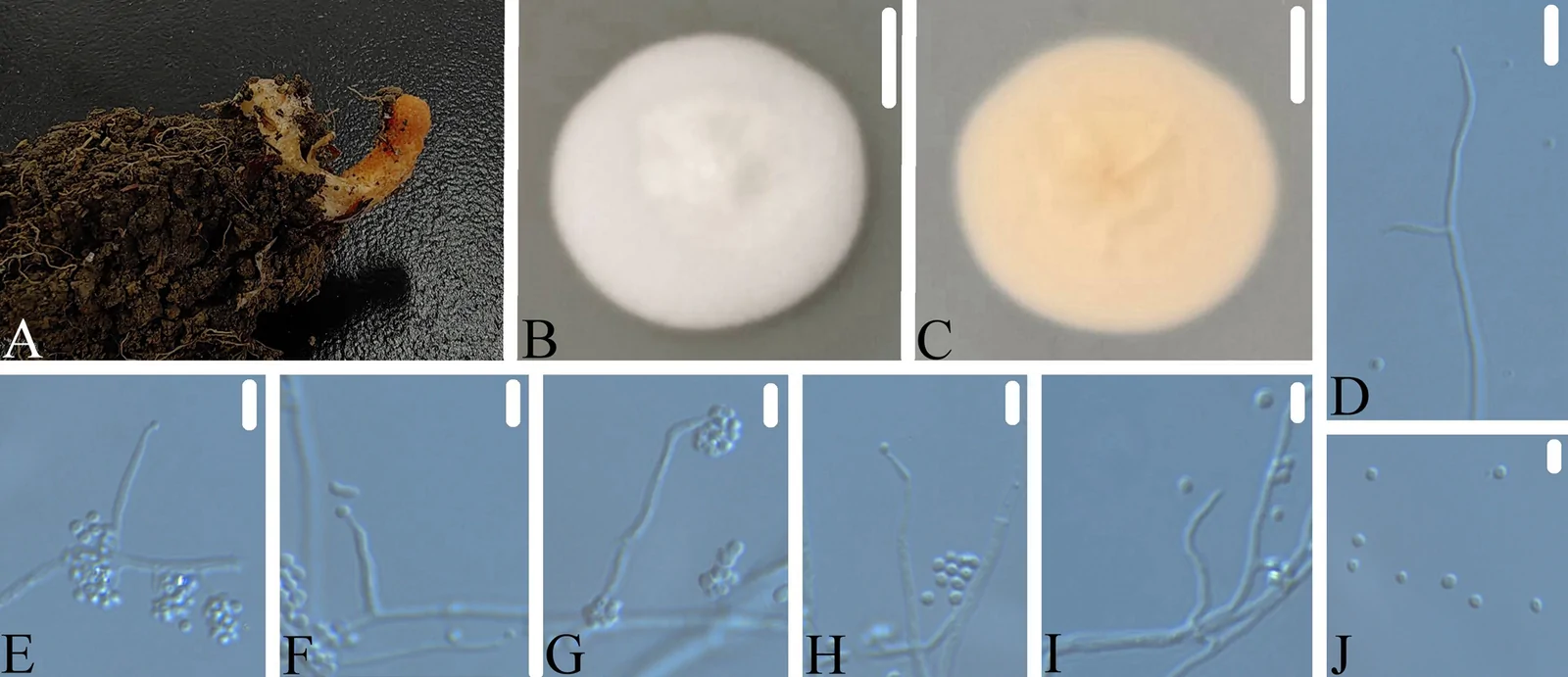
*Tolypocladium guizhouense*. **A**. *Cordyceps* sp. substrate; **B, C**. PDA culture plate showing top (**B**) and reverse (**C**) sides of the colony; **D–J**. Phialides and conidia. Scale bars: 10 mm (**B, C**); 10 μm (**D–J**).

##### Type.

China • Guizhou Province, Tongren City, Jiangkou County, Fanjing Mountain (27°50'56.98"N, 108°46'52.28"E). On *Cordyceps* sp., 1 May 2023, Wanhao Chen, GZAC TR0505 (holotype), TR05051 (ex-type).

##### Description.

Colonies on PDA attaining a diameter of 30–31 mm after 14 days at 25 °C. Colonies white, circular, consisting of a basal felt and cottony, floccose overgrowth, reverse pale yellow to light orange. Hyphae hyaline, branched, smooth-walled, 1.4–2.4 μm wide. Phialides usually curved, solitary, with a cylindrical basal portion, 12.1–26.3 × 1.6–2.2 μm (x̄ = 17.6 × 1.9 μm, n = 30), narrowing slightly. Conidia subglobose to ellipsoidal, 2.0–3.0 × 1.9–2.1 μm (x̄ = 2.3 × 2.0 μm, n = 30), single or aggregating in small orange-shaped heads at the apex of phialides. Chlamydospores not observed.

##### Substrate.

*Cordyceps* sp.

##### Habitat.

Litter layer.

##### Additional isotype examined.

China • Guizhou Province, Tongren City, Jiangkou County, Fanjing Mountain (27°50'56.98"N, 108°46'52.28"E). On *Cordyceps* sp., 1 May 2023, Wanhao Chen, TR05052 (living culture).

##### Note.

Molecular phylogenetic analysis of the concatenated dataset (Fig. 1) and NCBI BLASTn comparisons revealed that *Tolypocladium guizhouense* (TR05051 and TR05052) was grouped with *Tolypocladium* species. Phylogenetic analysis placed the species within a distinct clade sister to *T. pseudoalbum*, *T. tongrenense* and *T. zongqii*. In morphology, *Tolypocladium guizhouense* was distinguished from *T. pseudoalbum* by its absence of chlamydospores and by being found on a *Cordyceps* sp. substrate. *Tolypocladium guizhouense* was distinguished from *T. tongrenense* by its longer phialide (17.6 × 1.9 μm vs. 11.9 × 1.1 μm), being found on a *Cordyceps* sp. substrate and only one type of conidia. *Tolypocladium guizhouense* was distinguished from *T. zongqii* by its longer phialide (17.6 × 1.9 μm vs. 13.1 × 1.6 μm) and being found on a *Cordyceps* sp. The combined morphological characteristics, phylogenetic analyses, and PHI test (Fig. 2) provide conclusive support for the establishment of *T. guizhouense* as a new species.

#### Tolypocladium tongrenense

Taxon classificationFungiSordariomycetesOphiocordycipitaceae

W.H. Chen, Y.F. Han & J.D. Liang sp. nov.

CCA2AD46-2A07-57EC-B052-0285B60B49C2

Index Fungorum: IF905335

Fig. 4

##### Etymology.

Refers to the location, Tongren City, where the fungus was first discovered.

**Figure 4.**
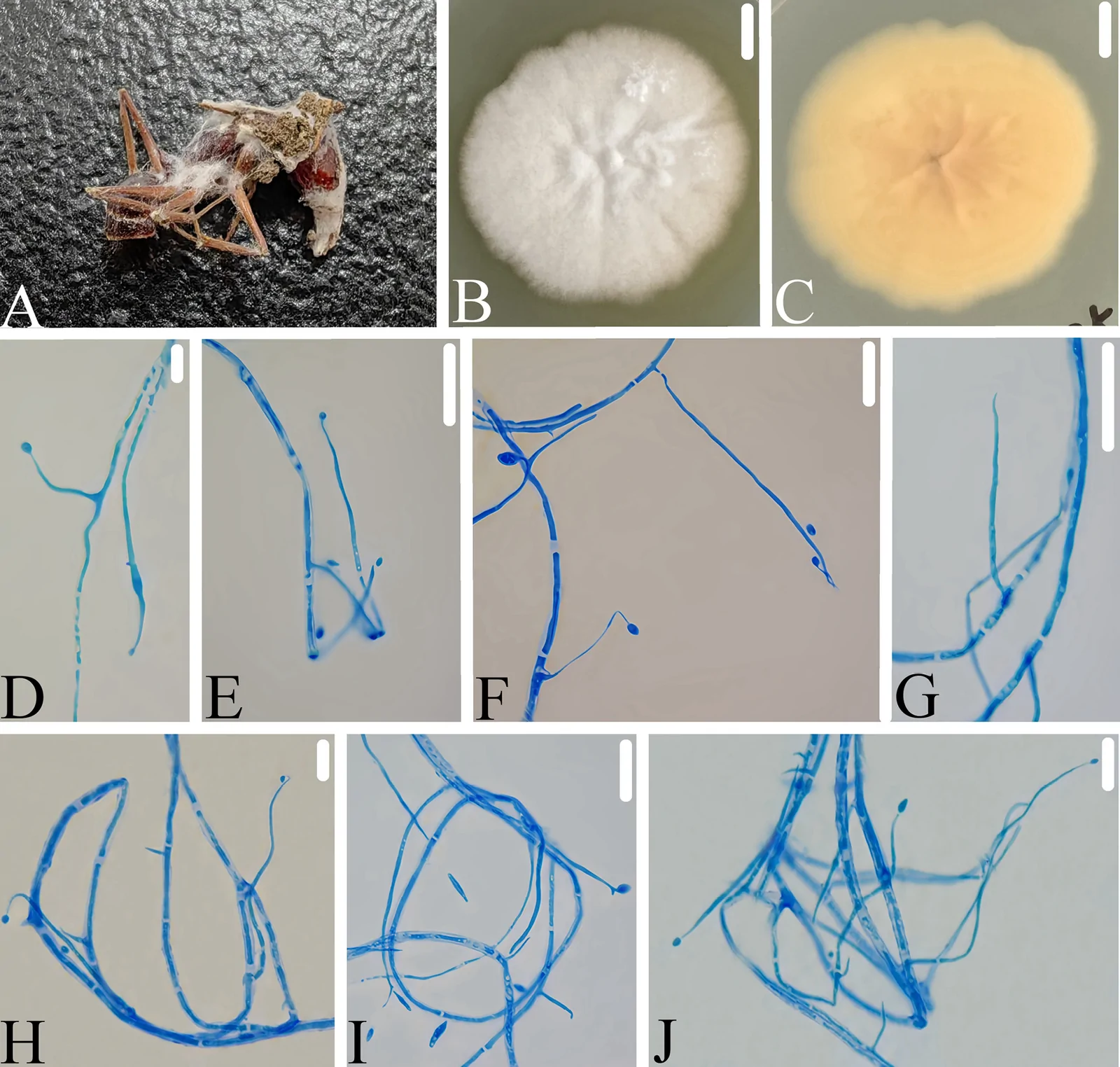
*Tolypocladium tongrenense*. **A**. Ant host; **B, C**. PDA culture plate showing top (**B**) and reverse (**C**) sides of the colony; **D–J**. Phialides and conidia. Scale bars: 10 mm (**B, C**); 10 μm (**D–J**).

##### Type.

China • Guizhou Province, Tongren City, Jiangkou County, Fanjing Mountain (27°50'56.98"N, 108°46'52.28"E). On a dead ant, 1 May 2023, Wanhao Chen, GZAC TR0520 (holotype), TR05201 (ex-type).

##### Description.

Colonies on PDA attaining a diameter of 49–52 mm after 14 days at 25 °C. Colonies white, circular, consisting of a basal felt and cottony, floccose overgrowth, reverse yellow to light orange, with radial striations. Hyphae hyaline, branched, smooth-walled, 0.8–2.4 μm wide. Phialides usually curved, solitary, with a cylindrical basal portion, 8.4–17.8 × 0.8–1.2 μm (x̄ = 11.9 × 1.1 μm, n = 30), narrowing slightly. Conidia in two types, ellipsoidal, 1.7–2.8 × 1.2–1.5 μm (x̄ = 2.1 × 1.3 μm, n = 30), or occasionally clavate, 3.6–5.6 × 1.2–1.7 μm (x̄ = 4.0 × 1.4 μm, n = 30), single. Chlamydospores not observed.

##### Substrate.

Ant (*Formicidae*).

##### Habitat.

Litter layer.

##### Additional isotype examined.

China • Guizhou Province, Tongren City, Jiangkou County, Fanjing Mountain (27°50'56.98"N, 108°46'52.28"E). On a dead ant, 1 May 2023, Wanhao Chen, TR05202 (living culture).

##### Note.

Molecular phylogenetic analysis of the concatenated dataset (Fig. 1) and NCBI BLASTn comparisons revealed that *Tolypocladium tongrenense* (TR05201 and TR05202) was grouped with *Tolypocladium* species. Phylogenetic analysis placed the species within a distinct clade sister to *T. guizhouense*, *T. pseudoalbum* and *T. zongqii*. In morphology, *Tolypocladium tongrenense* was distinguished from *T. guizhouense* by shorter phialide (11.9 × 1.1 μm vs. 17.6 × 1.9 μm) and an ant host. *Tolypocladium tongrenense* was distinguished from *T. pseudoalbum* by its shorter phialide (11.9 × 1.1 μm vs. 12.3–48.5 × 1.0–2.0 μm) and two types of conidia. *Tolypocladium tongrenense* was distinguished from *T. zongqii* by its shorter phialide (11.9 × 1.1 μm vs. 13.1 × 1.6 μm) and two types of conidia. The combined morphological characteristics, phylogenetic analyses and PHI test (Fig. 2) provide conclusive support for establishing *T. tongrenense* as a new species.

#### Tolypocladium zongqii

Taxon classificationFungiSordariomycetesOphiocordycipitaceae

W.H. Chen, Y.F. Han & J.D. Liang sp. nov.

93ECA613-2DDF-5F3A-A7FE-AAC738C02E38

Index Fungorum: IF905336

Fig. 5

##### Etymology.

In honor of Prof. Zongqi Liang, for his support and guidance in arthropod pathogenic fungi research.

**Figure 5.**
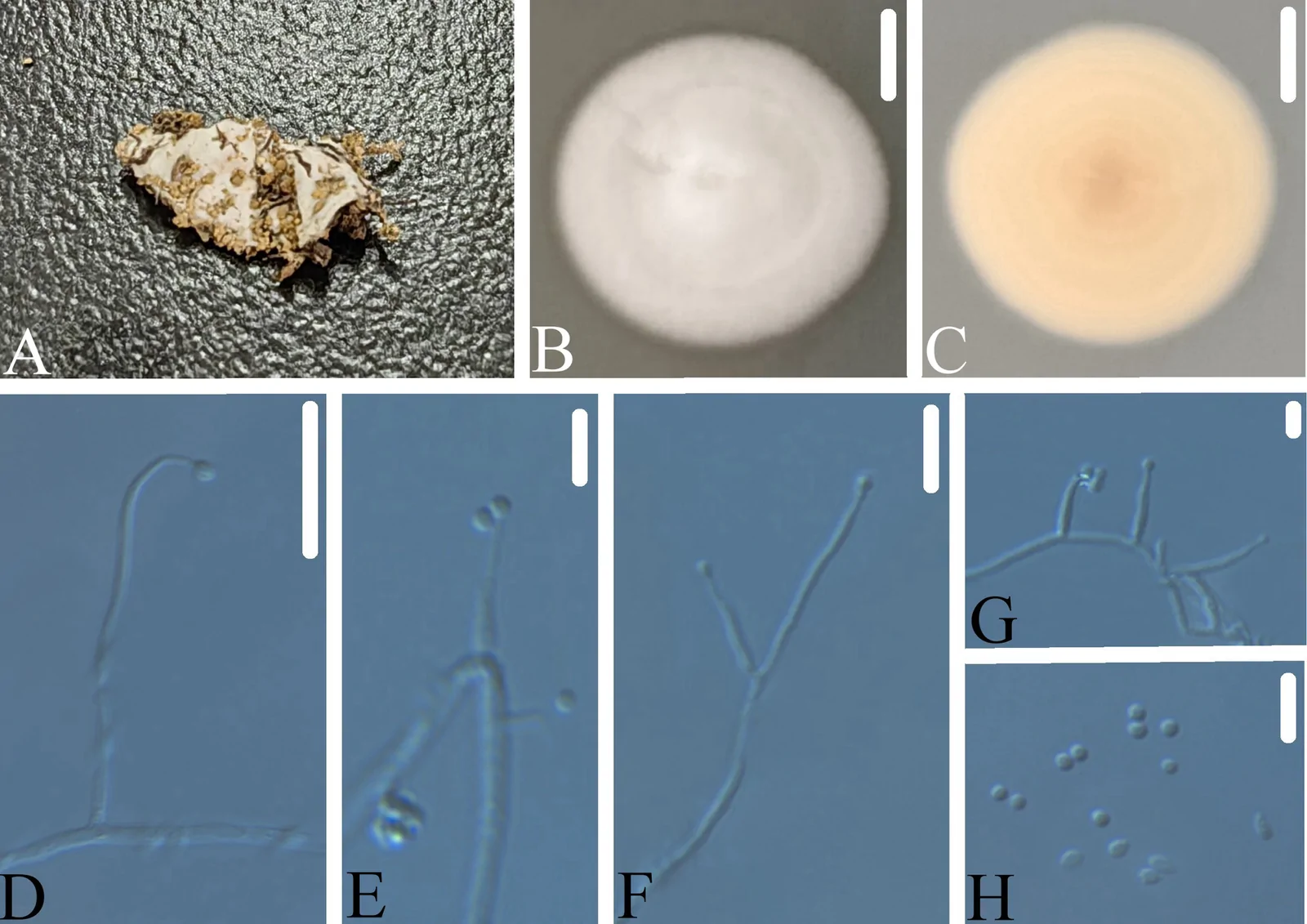
*Tolypocladium zongqii***A**. Pupa host; **B, C**. PDA culture plate showing top (**B**) and reverse (**C**) sides of the colony; **D–H**. Phialides and conidia. Scale bars: 10 mm (**B, C**); 10 μm (**D–H**).

##### Type.

China • Guizhou Province, Tongren City, Jiangkou County, Fanjing Mountain (27°50'56.98"N, 108°46'52.28"E). On a pupa, 1 May 2023, Wanhao Chen, GZAC TR0530 (holotype), TR05301 (ex-type).

##### Description.

Colonies on PDA attaining a diameter of 31–33 mm after 14 days at 25 °C. Colonies white, circular, consisting of a basal felt and cottony, floccose overgrowth, reverse yellow to light brown. Hyphae hyaline, branched, smooth-walled, 1.2–1.3 μm wide. Phialides usually curved, solitary, with a cylindrical basal portion, 9.4–23.0 × 1.5–2.0 μm (x̄ = 13.1 × 1.6 μm, n = 30), narrowing slightly. Conidia subglobose to ellipsoidal, 2.1–4.0 × 1.7–2.0 μm (x̄ = 2.7 × 1.8 μm, n = 30), single. Chlamydospores not observed.

##### Substrate.

Pupa (*Lepidoptera*).

##### Habitat.

Litter layer.

##### Additional isotype examined.

China • Guizhou Province, Tongren City, Jiangkou County, Fanjing Mountain (27°50'56.98"N, 108°46'52.28"E). On a pupa, 1 May 2023, Wanhao Chen, TR05302 (living culture).

##### Note.

Molecular phylogenetic analysis of the concatenated dataset (Fig. 1) and NCBI BLASTn comparisons revealed that *Tolypocladium zongqii* (TR05301 and TR05302) was grouped with *Tolypocladium* species. Phylogenetic analysis placed the species within a distinct clade sister to *T. guizhouense*, *T. pseudoalbum* and *T. tongrenense*. In morphology, *Tolypocladium zongqii* was distinguished from *T. guizhouense* by shorter phialide (13.1 × 1.6 μm vs. 17.6 × 1.9 μm) and a pupa host. *Tolypocladium zongqii* was distinguished from *T. pseudoalbum* by its absence of chlamydospores and a pupa host. *Tolypocladium zongqii* was distinguished from *T. tongrenense* by its longer phialide (13.1 × 1.6 μm vs. 11.9 × 1.1 μm) and only one type of conidia. The combined morphological characteristics, phylogenetic analyses, and PHI test (Fig. 2) provide conclusive support for the establishment of *T. zongqii* as a new species.

### Taxonomic key to distinguish the species of *Tolypocladium*

**Table:** 

1	Sexual state observed	**2**
–	Sexual state not observed	**28**
2	Perithecia superficial or half-immersed	**3**
–	Perithecia completely immersed	**4**
3	Perithecia pyriform, relatively larger, 520–550 × 260–280 μm, asci relatively larger, 400–450 × 7–7.5 μm, part spores 2.5–3.0 × 3.0 μm, on cicada nymphs, stromata relatively longer, 14 cm long	***T. inegoense***
–	Perithecia ovoid, relatively smaller, 320–380 × 220–280 μm, asci cylindrical, smaller, 240–250 × 6 μm, not dissociate into part spores, on *Elaphomyces*, stromata shorter, 3.5–4.5 cm long	***T. ramosum***
4	Perithecia ellipsoid, subglobose to ovoid	**5**
–	Perithecia ampullaceous	**27**
5	From multiple substrate/host (beetle or moth larvae, Larvae of *Scarabaeidae* (sexual morph); soil, humus, *Picea glauca*, roots of *Picea mariana*, the surface of *Mycobates* sp. (Acari, *Mycobatidae*), the sclerotium of *Ophiocordyceps gracilis* (asexual morph)	***T. inflatum***
–	From simple substrate/host	**6**
6	On beetle or unidentified host	**7**
–	On *Elaphomyces*	**10**
7	On the unidentified host, asci relatively wider, 10–15 μm	***T. cucullae***
–	On beetle, asci narrower than 10 μm	**8**
8	Stromata was connected to the host through a yellowish rhizomorph-like structure	***T. fumosum***
–	Stromata arising directly from the host, never rhizomorphic	**9**
9	Part spores short cylindrical, truncate at both ends, 3–5 × 1.5–2 μm	***T. paradoxum***
–	Part spores very short, almost cuboid in side view, without flattened ends, 1.5–2.5 × 1.5–1.7 μm	***T. toriharamontanum***
10	Stromata clavate, the fertile part not abruptly enlarged from the stipe	**11**
–	Stromata capitate, the fertile part spherical, oval or cylindrical abruptly enlarged from the stipe	**18**
11	Stromata size relatively larger, 10–12 cm long	***T. jezoense***
–	Stromata size < 10 cm	**12**
12	Part spores articulate, moniliform	***T. szemaoense***
–	Part spores cylindrical	**13**
13	Stromata was connected to the host through a rhizomorph-like structure	**14**
–	Stromata arising directly from the host, never rhizomorphic	**15**
14	Fertile part yellowish-green when young, turning olive-green as it matures, perithecia relatively smaller, 480–590 × 195–235 μm	***T. bacillisporum***
–	Fertile part reddish brown to olivaceous brown, perithecia larger, 600–800 × 250–500 μm	***T. ophioglossoides***
15	Fertile part black, yellow black, dark chestnut brown when dried	**16**
–	Fertile part pale bluish to grayish blue	***T. valvatistipitatum***
16	Perithecia ≤ 700 μm long	***17***
–	Perithecia > 700 μm long (750–1000 × 250–300 μm)	***T. tenuisporum***
17	Perithecia relatively narrower, 567–697 × 206–248 μm, part spores smaller, 2–5 × 1.5–2 μm, stromata 1.5–3 cm long	***T. flavonigrum***
–	Perithecia relatively wider, 500–700 × 250–350 μm, part spores larger, 10–18 × 2.5–4 μm, stromata 2.5–7 cm long	***T. japonicum***
18	Perithecia larger	**19**
–	Perithecia smaller, 400 × 250 μm	***T. virens***
19	Stromata 12 cm long, part spores very long, 40–65 μm long	***T. longisegmentatum***
–	Stromata shorter than 12 cm, part spores < 40 μm long	**20**
20	Part spores ≤ 8 μm long	**21**
–	Part spores > 8 μm long	**23**
21	Asci shorter than 300 μm (240–300 × 7–8 μm), perithecia relatively smaller (450–540 × 230–260 μm)	***T. intermedium***
–	Asci longer than 300 μm, perithecia larger	**22**
22	Stipe slender, 0.5–1.0 mm thick, yellowish green to olivaceous, stromata shorter, 1.5–2.5 cm long, part spores, 2–5 × 1.5–2 μm	***T. fractum***
–	Stipe 1–5 mm thick, dark brown, smooth or furfuraceous, stromata 5–7 cm long, part spores longer, 3–8 × 2 μm	***T. valliforme***
23	Perithecia < 550 μm long (480–540 μm)	***T. delicatistipitatum***
–	Perithecia > 550 μm long	**24**
24	Part spores < 15 μm long (8–11 μm)	***T. miomoteanum***
–	Part spores ≥ 15 μm long	**25**
25	Part spores < 3 μm wide	**26**
–	Part spores ≥ 3 μm wide (3.0–4.5 μm)	***T. inusitaticapitatum***
26	Fertile part olive-brown to olive-black, perithecia relatively larger, 650–950 × 250–420 μm, asci wider, 350–540 × 10–12 μm, part spores cylindrical or somewhat fusoid, 8–25 × 2.5–3 μm	***T. capitatum***
–	Fertile part purple-brown, blacker when older, perithecia smaller, 600–750 × 200–300 μm, asci slender, 350–500 × 8–10 μm, part spores filiform, spindle-shaped, 15–20 × 2–3 μm	***T. rouxii***
27	Perithecia relatively shorter, 520–740 × 300–330 μm, part spores cylindrical, 3–7 × 2–3 μm, on cicada nymphs	***T. dujiaolongae***
–	Perithecia relatively longer, 900–930 × 220–250 μm, part spores fusoid, 16–18 × 3 μm, on *Elaphomyces*	***T. minazukiense***
28	From multiple substrate/host	**29 (*T. album* , *T. cylindrosporum* , *T. inflatum* , *T. pustulatum* , *T. subparadoxum* )**
–	From only a type of substrate/host	**32**
29	Phialides cylindrical	**30**
–	Phialides ellipsoidal to subglobos	***T. cylindrosporum***
30	Colonies white, conidia globose to ovoid (phialides 3.5–10 × 1–1.5 μm, conidia 3.5 × 1.5–2.0 μm)	***T. album***
–	Colonies white to pale yellow, conidia ellipsoidal, globose or broadly ellipsoidal	**31**
31	Phialides 4–10 × 2–4 μm, conidia 2–3 × 1.5–2.5 μm	***T. pustulatum***
–	Phialides 5.4–40.1 × 0.9–1.8 μm, conidia larger, 2.6–6.5 × 1–2.9 μm	***T. subparadoxum***
32	From substrate	**33**
–	On arthropod or bdelloid rotifers	**53**
33	Substrate is not fungus	**34**
–	Substrate is fungus	**50**
34	From plant tissue	**35 (*T. amazonense* , *T. endophyticum* , *T. ovalisporum* , *T. subtropicale* , *T. trecense* , *T. tropicale* )**
–	From soil or air	**40 (*T. geodes* , *T. globosum* , *T. microsporum* , *T. nubicola* , *T. pennsylvanicum* , *T. pseudoalbum* , *T. sobaeksanense* , *T. terrae* , *T. terricola* , *T. tundrense* , *T. yunnanense* )**
35	Conidia relatively smaller (globose,1.3 μm diam)	***T. endophyticum***
–	Conidia larger, diam > 1.3 μm	**36**
36	Conidia > 4 μm long (4.5–9.0 × 2.5–3.5 μm)	***T. ovalisporum***
–	Conidia < 4 μm long	**37**
37	Phialide > 4.6 μm long	**38**
–	Phialide < 4.6 μm long	**39**
38	Phialides 3.5–19 × 1–2.5 μm, conidia globose to ellipsoidal, 2 ± 0.4 μm	***T. subtropicale***
–	Phialides 6.5–13 × 0.9–2.4 μm, conidia globose, 2 ± 0.6 μm	***T. trecense***
39	Phialides 4.6 ± 1.2 × 1.5 ± 0.3 μm, conidia spherical, larger, 2.1–2.2 μm diam	***T. amazonense***
–	Phialide 4.6 ± 1.5 μm, conidia spherical, relatively smaller, 1.5 ± 0.1 μm diam	***T. tropicale***
40	From air	***T. pennsylvanicum***
–	From soil	**41**
41	Phialides cylindrical	**42**
–	Phialides subglobose or ellipsoidal	**48**
42	Conidia asymmetrically flattened, with a minute apical	***T. microsporum***
–	Conidia ellipsoidal, globose or broadly ellipsoidal	**43**
43	Colonies white to light beige	***T. globosum***
–	Colonies white or pale yellow or whitish to orange-yellow	**44**
44	Colonies white	**45**
–	Colonies pale yellow or whitish to orange-yellow	**47**
45	Conidia subglobose or broadly obovoid	***T. geodes***
–	Conidia globose	**46**
46	Phialides subcylindrical, 12.2 ± 4.7 × 1.7 ± 0.4, conidia 2.1 ± 0.1 μm	***T. sobaeksanense***
–	Phialides cylindrical, 6.5–13 × 0.9–2.4, conidia 2.0 ± 0.6 μm	***T. terrae***
47	Colonies white or pale yellow, phialide cylindrical, 12.3–48.5 × 1.0–2.0 μm, conidia 1.8–3.4 × 1.3–1.9 μm	***T. pseudoalbum***
–	Colonies white to whitish to orange-yellow, phialides 7.6–62.6 × 0.9–2.3 μm, conidia 1.2–2.4 × 0.9–1.9 μm)	***T. yunnanense***
48	Conidia only one type	**49**
–	Conidia two types (microconidia ellipsoidal or reniform, 2.3–4.2 × 1.3–2.3 μm, macroconidia: cylindrical, 10 × 2.4 μm)	***T. tundrense***
49	Phialides relatively longer, 4.4–7.8 × 1.5–2.7 μm, conidia cylindrical, 2.6–4.1 × 0.8–1.3 μm, colonies white to pale cream	***T. nubicola***
–	Phialides shorter, 2.8–3.5 × 2.0–3.0 μm, conidia broadly oval, 2.5–3 × 2.0–2.5 μm, colonies white	***T. terricola***
50	On *Elaphomyces*	***T. guangdongense***
–	On *Cordyceps* sensu lato	**51**
51	On *Cordyceps* sp.	***T. guizhouense***
–	From *Ophiocordyceps sinensis*	**52**
52	Conidia reniform, 1.0–3.2 × 0.7–1.6 μm, phialides 3.4–10.6 × 1.1–3.8 μm	***T. reniformisporum***
–	Conidia spherical, 1.4–3.6 μm diam, phialides 7.6–19.4 × 2.9–3.6 μm	***T. sinense***
53	On arthropod	**54**
–	On bdelloid rotifers	***T. trigonosporum***
54	On ant, phialide 11.9 × 1.1 μm, conidia ellipsoidal to clavate	***T. tongrenense***
–	On pupa, phialide 13.1 × 1.6 μm, conidia subglobose to ellipsoidal	***T. zongqii***

### *Clavicipitaceae* Rogerson, Mycologia 62(5): 900 (1970)

#### Metapochonia

Taxon classificationFungiSordariomycetesClavicipitaceae

Kepler, S.A. Rehner & Humber, in Kepler, Humber, Bischoff and Rehner, Mycologia 106(4): 820 (2014)

39218140-35CA-57CB-BCFF-701563A97498

##### Type species.

*Metapochonia suchlasporia* (W. Gams & Dackman) Kepler, Rehner and Humber 2014.

##### Note.

Kepler et al. (2014) established the genus *Metapochonia*, designating *M. suchlasporia* (W. Gams & Dackman) Kepler, S.A. Rehner and Humber as the type species, along with four other species. The genus has since been expanded and currently comprises eleven species. Among these, *Metapochonia parasitica* (G.L. Barron) Labuda, Bernr. & Kubátová was transferred from the genus *Tolypocladium*. In the present study, we propose a new combination, also derived from *Tolypocladium*.

#### Metapochonia lignicola

Taxon classificationFungiSordariomycetesClavicipitaceae

(G.L. Barron) W.H. Chen, Y.F. Han & J.D. Liang comb. nov.

97F8D935-AFC7-5947-A76B-6A3E27E36154

Index Fungorum: IF905337

107333

Tolypocladium lignicola G.L. Barron, Canad. J. Bot. 61(10): 2566 (1983). Basionym.

##### Type.

Canada • Ontario, Wellington County Forest, Tract, from rotting wood collected in Little Tract, in bdelloid rotifers, September 1982, slides in herbarium (OAC No. 10828), ex-type, CBS 489.83.

##### Description.

See Barron 1983.

##### Note.

Barron (1983) reported a new species *Tolypocladium lignicola*, from Canada. However, in our phylogenetic tree (Fig. 1), *Tolypocladium lignicola* (CBS 489.83) grouped with *Metapochonia* s. str., rather than with *Tolypocladium* in *Ophiocordycipitaceae*. Furthermore, NCBI BLASTn comparisons of the sequences of strain CBS 489.83 showed that it is closely related to *Metapochonia*. Thus, the new combination is proposed and named as *M. lignicola*.

## Discussion

A comprehensive phylogenetic reassessment of the genus *Tolypocladium* was carried out using a five-locus dataset (ITS, LSU, *rpb1*, *rpb2*, and *tef*-1α) and expanded taxon sampling that included newly collected specimens from Guizhou Province, China. Our results revealed that *Tolypocladium* as traditionally circumscribed, is polyphyletic, with species distributed across three distinct families of *Hypocreales*: *Ophiocordycipitaceae*, *Cordycipitaceae*, and *Clavicipitaceae*. Based on these phylogenetic findings, a new genus, *Tolypocladiopsis* is proposed; three new species, *Tolypocladium guizhouense*, *T. tongrenense*, and *T. zongqii*, are described; and two new combinations, *Tolypocladiopsis extinguens* and *Metapochonia lignicola*, are introduced. The generic delimitation of *Tolypocladium* is thereby refined, and the phylogenetic placement of several previously problematic taxa is clarified.

Dong et al. (2022) showed that *Tolypocladium extinguens* clustered outside the core *Tolypocladium* s. str. in their phylogenetic tree but did not formally propose a new genus, likely due to the limited taxon sampling. Similarly, Quandt et al. (2014) synonymized *Elaphocordyceps* and *Chaunopycnis* under *Tolypocladium* following the “One Fungus-One Name” principle, while acknowledging that some species might eventually require reclassification as more molecular data become available. Our study provides robust phylogenetic evidence (Fig. 1) to resolve these long-standing ambiguities, and provides the taxonomic key to distinguish the species of *Tolypocladium*.

The new genus *Tolypocladiopsis* is recovered as a distinct monophyletic clade within *Cordycipitaceae* with strong ML bootstrap support (99%) but relatively low Bayesian posterior probability (PP < 0.95). The single-gene phylogenetic trees in which the ex-type strain of *Tolypocladiopsis* is represented (ITS and LSU; see Suppl. materials 2, 4) consistently recover this lineage as distinct from its related genera, further supporting its recognition as an independent genus. We attribute the weak BI support to several factors. The ITS and LSU regions, which are the only loci currently available for the ex-type strain CBS 345.77 of *Tolypocladiopsis extinguens*, contain a limited number of phylogenetically informative sites for this particular branch within *Cordycipitaceae*. The absence of protein-coding gene sequences (*rpb1*, *rpb2*, and *tef*-1α) for this critical strain likely reduces the overall phylogenetic signal, as these loci generally provide greater resolution for deep and moderately deep nodes in *Hypocreales*. The availability of only a single strain precludes the inclusion of multiple representatives that could strengthen branch support through increased taxon sampling. Despite the moderate BI value, the recognition of *Tolypocladiopsis* is robustly justified by: its consistent recovery as a distinct monophyletic lineage in both concatenated and single-gene analyses; its stable placement within *Cordycipitaceae*, clearly separated from *Ophiocordycipitaceae* where *Tolypocladium* s. str. resides; and its unique morphological features (paecilomyces-like conidiophores, ellipsoidal to subglobose, sometimes kidney-shaped conidia) that distinguish it from all currently recognized genera in *Cordycipitaceae*.

Our phylogeny shows that *Tolypocladium* s. str. (including the type species, *T. inflatum* and its close relatives) forms a well-supported monophyletic clade within the *Ophiocordycipitaceae*. However, two species historically assigned to *Tolypocladium* fall outside this clade: *T. extinguens* (*Cordycipitaceae*) and *T. lignicola* (*Clavicipitaceae*). These findings are consistent with the growing recognition that morphological convergence, particularly in the simple, reduced conidiogenous structures typical of tolypocladium-like anamorphs, often obscures evolutionary relationships within *Hypocreales* (Sung et al. 2007; Kepler et al. 2014).

Our phylogenetic analysis firmly places *Tolypocladium extinguens* within *Cordycipitaceae*, where it is phylogenetically placed in close proximity to genera such as *Dimorphspora*, *Flavocillium*, *Gamszarea*, and *Parengyodontium*. This result is unexpected because the morphological features of *T. extinguens*, paecilomyces-like conidiophores, swollen phialides with bent necks, and ellipsoidal to subglobose conidia, are typical of *Tolypocladium* (Samson and Soares Jr 1984). Nevertheless, our molecular data unequivocally reject its placement in *Ophiocordycipitaceae*. The new genus *Tolypocladiopsis* is therefore proposed to accommodate this species. The establishment of *Tolypocladiopsis* adds to the growing list of cordycipitaceous genera with tolypocladium-like anamorphs and highlights the need for integrated morphological and molecular approaches in hypocrealean systematics.

*Metapochonia lignicola* is a new combination involving a species originally described as *Tolypocladium lignicola* from rotting wood parasitizing bdelloid rotifers (Barron 1983). Our phylogenetic analysis places this species within the genus *Metapochonia (Clavicipitaceae)*, a finding consistent with the observation that several tolypocladium-like species associated with rotifers or nematodes actually belong to *Clavicipitaceae* rather than *Ophiocordycipitaceae* (Kepler et al. 2014). This result is not entirely surprising, as *Metapochonia* was originally segregated from *Tolypocladium* based on molecular phylogenetic evidence, and *M. parasitica* (G.L. Barron) Labuda et al. (formerly *T. parasiticum* G.L. Barron) was previously transferred to *Metapochonia* (Kepler et al. 2014). Our study adds a second rotifer-associated species to *Metapochonia*, suggesting that mycoparasitism and nematode/rotifer parasitism may have evolved multiple times across different hypocrealean families.

In addition, we describe three new species of *Tolypocladium* s. str. from Fanjing Mountain, Guizhou Province. The discovery of three new *Tolypocladium* species from a single locality in Guizhou Province highlights the high, yet underexplored, diversity of entomopathogenic and fungicolous fungi in southwestern China. Fanjing Mountain, a UNESCO (United Nations Educational, Scientific and Cultural Organization) World Heritage site, possesses high biodiversity and may harbor further undescribed fungal lineages (Wijayawardene et al. 2021). Our study underscores the importance of continued field surveys and integrative taxonomic approaches in this region.

Notably, *Metarhiziopsis* D.W. Li, R.S. Cowles & Vossbr. was originally assigned to *Clavicipitaceae* based on its sporodochial conidiomata and ecological association with scale insects (Li et al. 2008). However, our phylogenetic analysis unequivocally places this lineage within *Ophiocordycipitaceae*, where it forms a sister relationship with *Tolypocladium* s. str. (Fig. 1). In addition, the genera *Helicocollum* Luangsa-ard, Mongkols., Noisrip. & Thanakitp. and *Polymicrospora* Hong Yu bis, Z.Q. Wang, Z.Li Yang & J.Mei Ma form an independent clade and do not belong to *Clavicipitaceae*. Similarly, *Neobaryopsis* Flakus, Etayo, Kukwa & Rodr. Flakus forms a distinct clade outside *Cordycipitaceae*. Additionally, a close relationship between *Polymicrospora* and *Purpureocillium* Luangsa-ard, Hywel-Jones, Houbraken & Samson has been reported in Wang et al. (2025). Furthermore, *Hantamomyces aloidendri* Crous groups together with species of *Paraisaria* Samson & B.L. Brady, whereas species of *Torrubiellomyces* J.P.M. Araújo & de Bekker cluster with those of *Perennicordyceps* Matočec & I. Kušan, *Pleurocordyceps* Y.J. Yao, Y.Hui Wang, S. Ban, W.J. Wang, Yi Li, Ke Wang & P.M. Kirk, *Polycephalomyces* Kobayasi and to form a separate lineage (Fig. 1), with the latter genera all belonging to *Polycephalomycetaceae*.

It is also worth noting that, despite the expanded taxon sampling in this study, the phylogenetic relationships among several genera within *Ophiocordycipitaceae* remain poorly resolved, and some traditionally recognized genera do not form robust monophyletic lineages. This observation suggests that the generic delimitation within *Ophiocordycipitaceae* is more complex than previously appreciated. The correct taxonomic placement of these genera remains uncertain, and further phylogenetic analyses incorporating additional molecular markers (e.g., β-tubulin, act, or genomic-scale data), broader taxon sampling, and comprehensive morphological and ecological assessments are required to fully resolve their positions. Our study thus provides a refined framework for *Tolypocladium* while also highlighting outstanding questions that warrant future investigation.

## Supplementary Material

XML Treatment for Tolypocladiopsis

XML Treatment for Tolypocladiopsis extinguens

XML Treatment for Tolypocladium guizhouense

XML Treatment for Tolypocladium tongrenense

XML Treatment for Tolypocladium zongqii

XML Treatment for Metapochonia

XML Treatment for Metapochonia lignicola

## Additional information

### Artificial Intelligence (AI) use

The authors accept full responsibility for the content of the manuscript, including the disclosure of any use of AI.

No AI tools were used in the preparation of this manuscript.

### Author ORCIDs

W.-H. Chen https://orcid.org/0000-0001-7240-6841

H.-L. Shu https://orcid.org/0009-0003-1905-595X

D. Li https://orcid.org/0009-0009-0256-6749

Q. Zeng https://orcid.org/0009-0005-4978-7371

J.-D. Liang https://orcid.org/0000-0002-3939-3900

N.N. Wijayawardene https://orcid.org/0000-0003-0522-5498

Y.-F. Han https://orcid.org/0000-0002-8646-3975

J.-H. Zhao https://orcid.org/0000-0003-2972-382X

### Data availability

All of the data that support the findings of this study are available in the main text or Supplementary Information.

## Supplementary materials

Supplementary material 1 Dataset for fig. 1 Data type fas https://binary.pensoft.net/file/1808116This dataset is made available under the Open Database License (http://opendatacommons.org/licenses/odbl/1.0/). The Open Database License (ODbL) is a license agreement intended to allow users to freely share, modify, and use this Dataset while maintaining this same freedom for others, provided that the original source and author(s) are credited. Wan-Hao Chen, Chuan-Xuan Xiong, Hui-Lin Shu, Dan Li, Qian Zeng, Jian-Dong Liang, Nalin N. Wijayawardene, Yan-Feng Han, Jie-Hong Zhao

Supplementary material 2 Phylogram retrieved from IQTREE of the new species and Tolypocladium species using ITS sequences Data type jpg https://binary.pensoft.net/file/1808117This dataset is made available under the Open Database License (http://opendatacommons.org/licenses/odbl/1.0/). The Open Database License (ODbL) is a license agreement intended to allow users to freely share, modify, and use this Dataset while maintaining this same freedom for others, provided that the original source and author(s) are credited. Wan-Hao Chen, Chuan-Xuan Xiong, Hui-Lin Shu, Dan Li, Qian Zeng, Jian-Dong Liang, Nalin N. Wijayawardene, Yan-Feng Han, Jie-Hong Zhao

Supplementary material 3 Alignment used for fig. S1 Data type fas https://binary.pensoft.net/file/1808118This dataset is made available under the Open Database License (http://opendatacommons.org/licenses/odbl/1.0/). The Open Database License (ODbL) is a license agreement intended to allow users to freely share, modify, and use this Dataset while maintaining this same freedom for others, provided that the original source and author(s) are credited. Wan-Hao Chen, Chuan-Xuan Xiong, Hui-Lin Shu, Dan Li, Qian Zeng, Jian-Dong Liang, Nalin N. Wijayawardene, Yan-Feng Han, Jie-Hong Zhao

Supplementary material 4 Phylogram retrieved from IQTREE of the new species and Tolypocladium species using LSU sequences Data type jpg https://binary.pensoft.net/file/1808119This dataset is made available under the Open Database License (http://opendatacommons.org/licenses/odbl/1.0/). The Open Database License (ODbL) is a license agreement intended to allow users to freely share, modify, and use this Dataset while maintaining this same freedom for others, provided that the original source and author(s) are credited. Wan-Hao Chen, Chuan-Xuan Xiong, Hui-Lin Shu, Dan Li, Qian Zeng, Jian-Dong Liang, Nalin N. Wijayawardene, Yan-Feng Han, Jie-Hong Zhao

Supplementary material 5 Alignment used for fig. S2 Data type fas https://binary.pensoft.net/file/1808120This dataset is made available under the Open Database License (http://opendatacommons.org/licenses/odbl/1.0/). The Open Database License (ODbL) is a license agreement intended to allow users to freely share, modify, and use this Dataset while maintaining this same freedom for others, provided that the original source and author(s) are credited. Wan-Hao Chen, Chuan-Xuan Xiong, Hui-Lin Shu, Dan Li, Qian Zeng, Jian-Dong Liang, Nalin N. Wijayawardene, Yan-Feng Han, Jie-Hong Zhao

Supplementary material 6 Phylogram retrieved from IQTREE of the new species and Tolypocladium species using rpb1 sequences Data type jpg https://binary.pensoft.net/file/1808121This dataset is made available under the Open Database License (http://opendatacommons.org/licenses/odbl/1.0/). The Open Database License (ODbL) is a license agreement intended to allow users to freely share, modify, and use this Dataset while maintaining this same freedom for others, provided that the original source and author(s) are credited. Wan-Hao Chen, Chuan-Xuan Xiong, Hui-Lin Shu, Dan Li, Qian Zeng, Jian-Dong Liang, Nalin N. Wijayawardene, Yan-Feng Han, Jie-Hong Zhao

Supplementary material 7 Alignment used for fig. S3 Data type fas https://binary.pensoft.net/file/1808122This dataset is made available under the Open Database License (http://opendatacommons.org/licenses/odbl/1.0/). The Open Database License (ODbL) is a license agreement intended to allow users to freely share, modify, and use this Dataset while maintaining this same freedom for others, provided that the original source and author(s) are credited. Wan-Hao Chen, Chuan-Xuan Xiong, Hui-Lin Shu, Dan Li, Qian Zeng, Jian-Dong Liang, Nalin N. Wijayawardene, Yan-Feng Han, Jie-Hong Zhao

Supplementary material 8 Phylogram retrieved from IQTREE of the new species and Tolypocladium species using rpb2 sequences Data type jpg https://binary.pensoft.net/file/1808123This dataset is made available under the Open Database License (http://opendatacommons.org/licenses/odbl/1.0/). The Open Database License (ODbL) is a license agreement intended to allow users to freely share, modify, and use this Dataset while maintaining this same freedom for others, provided that the original source and author(s) are credited. Wan-Hao Chen, Chuan-Xuan Xiong, Hui-Lin Shu, Dan Li, Qian Zeng, Jian-Dong Liang, Nalin N. Wijayawardene, Yan-Feng Han, Jie-Hong Zhao

Supplementary material 9 Alignment used for fig. S4 Data type fas https://binary.pensoft.net/file/1808124This dataset is made available under the Open Database License (http://opendatacommons.org/licenses/odbl/1.0/). The Open Database License (ODbL) is a license agreement intended to allow users to freely share, modify, and use this Dataset while maintaining this same freedom for others, provided that the original source and author(s) are credited. Wan-Hao Chen, Chuan-Xuan Xiong, Hui-Lin Shu, Dan Li, Qian Zeng, Jian-Dong Liang, Nalin N. Wijayawardene, Yan-Feng Han, Jie-Hong Zhao

Supplementary material 10 Phylogram retrieved from IQTREE of the new species and Tolypocladium species using tef-1a sequences Data type jpg https://binary.pensoft.net/file/1808125This dataset is made available under the Open Database License (http://opendatacommons.org/licenses/odbl/1.0/). The Open Database License (ODbL) is a license agreement intended to allow users to freely share, modify, and use this Dataset while maintaining this same freedom for others, provided that the original source and author(s) are credited. Wan-Hao Chen, Chuan-Xuan Xiong, Hui-Lin Shu, Dan Li, Qian Zeng, Jian-Dong Liang, Nalin N. Wijayawardene, Yan-Feng Han, Jie-Hong Zhao

Supplementary material 11 Alignment used for fig. S5 Data type fas https://binary.pensoft.net/file/1808126This dataset is made available under the Open Database License (http://opendatacommons.org/licenses/odbl/1.0/). The Open Database License (ODbL) is a license agreement intended to allow users to freely share, modify, and use this Dataset while maintaining this same freedom for others, provided that the original source and author(s) are credited. Wan-Hao Chen, Chuan-Xuan Xiong, Hui-Lin Shu, Dan Li, Qian Zeng, Jian-Dong Liang, Nalin N. Wijayawardene, Yan-Feng Han, Jie-Hong Zhao
